# Defining and searching for structural motifs using DeepView/Swiss-PdbViewer

**DOI:** 10.1186/1471-2105-13-173

**Published:** 2012-07-23

**Authors:** Maria U Johansson, Vincent Zoete, Olivier Michielin, Nicolas Guex

**Affiliations:** 1 Vital-IT Group, SIB Swiss Institute of Bioinformatics, CH-1015, Lausanne, Switzerland; 2 Molecular Modelling Group, SIB Swiss Institute of Bioinformatics, CH-1015, Lausanne, Switzerland

## Abstract

**Background:**

Today, recognition and classification of sequence motifs and protein folds is a mature field, thanks to the availability of numerous comprehensive and easy to use software packages and web-based services. Recognition of structural motifs, by comparison, is less well developed and much less frequently used, possibly due to a lack of easily accessible and easy to use software.

**Results:**

In this paper, we describe an extension of DeepView/Swiss-PdbViewer through which structural motifs may be defined and searched for in large protein structure databases, and we show that common structural motifs involved in stabilizing protein folds are present in evolutionarily and structurally unrelated proteins, also in deeply buried locations which are not obviously related to protein function.

**Conclusions:**

The possibility to define custom motifs and search for their occurrence in other proteins permits the identification of recurrent arrangements of residues that could have structural implications. The possibility to do so without having to maintain a complex software/hardware installation on site brings this technology to experts and non-experts alike.

## Background

The three-dimensional structure of proteins has been an extensively studied topic for several decades. More than fifty years ago, Pauling and Corey described the two dominant forms of secondary structure, the α-helix and the β-sheet [1]. Subsequently, a variety of further patterns and regularities (*e.g.*, [2-4]) in protein structures have been found, that have proven useful in the context of protein structure determination and quality assessment of determined structures. During the last twenty years, increasingly sophisticated methods for secondary structure prediction [5,6], fold recognition and comparison (*e.g.*, FSSP [7], THREADER [8], FOLDFIT [9], and others [10-12] have been developed, followed by methods for fold classification, such as SCOP [13] and CATH [14]. More or less simultaneously, methods were developed for identifying and searching for structural similarities involving limited numbers of amino acid residues (*e.g.*, [15-19]), and more recently for the prediction of protein function, or functional groups, through recognition of geometrical patterns that involve small numbers of residues (*e.g.*, [18,20-25]).

Quite a few of the previously mentioned patterns and regularities in protein structures, and the associated methods for detecting them, in particular, have constraints and limitations that make them ill-suited to searching for general structural motifs, such as only dealing with sequentially compact fragments [15], only considering amino-acids that are conserved among homologous proteins [16], or restricted to small subsets of atom types (*i.e.,* N, Cα and C’ [17], Cα and Cβ [18], and Cα and pseudo atom at side-chain centre of gravity [19]). What is much more important from a practical usability perspective, however, is the fact that none of the mentioned methods have been well integrated into any comprehensive and widely available molecular graphics software package. Our purpose in this paper is to present the mechanisms and facilities whereby structural motifs can be defined and searched for using the freely available and well established modelling tool Swiss-PdbViewer [26], and to present illustrative examples of the types of information that can be obtained by doing so. In particular, the facilities for defining and searching for structural motifs now available in Swiss-PdbViewer include an interactive visual interface for defining structural motifs, and a machinery that is able to quickly search very large collections of structures for such motifs. Finally, to our knowledge, the presented structure-search machinery is the only one to permit arbitrary combinations of amino-acid-type constraints, secondary structure constraints, distance constraints, and sequence separation constraints.

The main reason for our interest in general (*i.e.*, sequentially non-contiguous) structural motifs, is the crucial role played by side-chains in the correct packing of proteins. In the context of structural protein modelling, slight differences in backbone conformations may accommodate entirely different combinations of side-chain conformations [27], and inadequate sampling of conformational space typically leads to suboptimal conformations being found, which in turn leads to a degradation of model quality as artificially loose protein cores are formed in order to leave more space for side-chains [28]. Side-chain conformations are not currently given sufficient consideration, and new approaches need to be pursued in order to make it possible to do so.

## Implementation

### Structural motifs

The notion of structural motifs has not been clearly defined, and finding a definition that is both precise and useful is not as simple as it might first appear. In essence, there are two main methods of quantifying structural similarity (of proteins). The first and most commonly used measure of similarity between two *n*-atom configurations, A and B, is the so-called root mean square deviation (rmsd), the value of which is obtained as:

(1) $$rmsd(A,B)=\sqrt{\frac{1}{n}\sum_{i=1}^{n}{\left(\left\|a_{i}-b_{i}\right\|\right)}^{2}}$$

where each ** *a* **_*i*_** *b* **_*i*_ ∈ ℜ^3^ corresponds to the three-component coordinate vector of atom *i* in *A* and *B*, respectively. A meaningful rmsd-value, however, requires that the atom-configurations *A* and *B* have been optimally superposed in a least-squares sense. However, such optimal super-positioning of atom-configurations requires an *O*(*n*) computational effort for configurations of *n* atoms in three-dimensional space (*e.g.,* see [29,30]). When searching for a structural motif of *k* sequentially non-adjacent residues in a protein structure *P*, comprising *m* residues in total, an rmsd-value may need to be calculated as described above for every subset of *k* residues drawn from the *m* residues of *P*. The total number of *k*-residue subsets that can be drawn from *m* residues is given by the expression:

(2) $$\left(\begin{array}{c} m\\ k \end{array}\right)=\frac{m(m-1)(m-2)\ldots (m-k+1)}{k!}=\frac{m!}{k!\left(m-k\right)!}\text{,}$$

and thus increases at least exponentially with respect to *k* (for *k* < ⌊m/2⌋) and as a *k*th-order polynomial with respect to *m*. Although the number of operations needed to compute one single rmsd-value grows linearly with motif size and would thus not be a limiting factor, the overall number of computations necessary to evaluate the superposition of a *k*-residue motif loosely defined with respect to sequence constraints onto all possible combinations of *k*-residues drawn from a structure can nonetheless become noticeable in practice.

Furthermore, the rmsd measure is also in itself problematic in the present context, because values implying a meaningful degree of molecular similarity vary with the number and type of amino acid residues or atoms being used, and is also quite sensitive to outliers. This problem has been addressed by several authors [31-33], but the solutions proposed tend to involve empirically determined parameters and/or probability distributions that depend on the number of atoms involved and the presence or absence of chemical bonds between said atoms. In contexts where the number and types of amino acid residues in motifs as well as the sequential distance between residues in motifs will be highly variable, and the collection of protein structures participating in the analysis is allowed to vary (the pdb database is itself a constantly changing entity) it appears that making effective use of the mentioned methods for judging rmsd-values would be difficult. These well known issues with the rmsd measure have also prompted the assessors of the CASP community to develop more robust metrics to judge the quality of models [34]. The reasons mentioned above prompted us to choose a different approach than rmsd to identify atom configurations that satisfy a motif specification.

The second of the two main methods of quantifying structural similarity uses matrices, ** *D* **^*A*^ and ** *D* **^*B*^, consisting of all internal distances between atoms in the two collections of *n* atoms *A* and *B*.

(3) $$D^{A}=\left[\begin{array}{ccc} d_{11}^{A} & \cdots & d_{1n}^{A}\\ \cdots & & \cdots \\ d_{n1}^{A} & \cdots & d_{\mathrm{nn}}^{A} \end{array}\right],D^{B}=\left[\begin{array}{ccc} d_{11}^{B} & \cdots & d_{1n}^{B}\\ \cdots & & \cdots \\ d_{n1}^{B} & \cdots & d_{\mathrm{nn}}^{B} \end{array}\right]\text{,}$$

where *d*^*A*^_*ij*_ = || ** *a* **_*i*_ – ** *a* **_*j*_ ||_2_ and *d*^*B*^_*ij*_ = || ** *b* **_*i*_ – ** *b* **_*j*_ ||_2_, for *i* = 1,…,*n* and *j* = 1,…,*n*. The matrices ** *D* **^*A*^ and ** *D* **^*B*^ are typically referred to as distance matrices. The distance matrix is a well known and frequently used concept in structural characterizations of proteins (*e.g.*, see [35,36]), and does in fact contain sufficient information to reconstruct the three-dimensional structure of each corresponding protein [37]. Given the two distance matrices ** *D* **^*A*^ and ** *D* **^*B*^, a measure of the structural similarity between the two configurations of atoms *A* and *B* expressed as a single number, is obtained by forming ** *D* ** = ** *D* **^*A*^ – ** *D* **^*B*^ and evaluating the expression:

(4) $$\sum_{i-1}^{n}\sum_{j=1}^{n}\left|D_{\mathrm{ij}}\right|\text{,}$$

The structural similarity measure so obtained has more or less the same shortcomings as rmsd-values when it comes to interpreting its meaning. In addition thereto, the computations implied by Eqs. (3) and (4) require at least *n*^2^ operations, and it is thereby computationally *more* expensive than calculating rmsd values, for large values of *n*.

Defining a motif through an upper limit on the similarity measure above or by an rmsd upper limit, cannot be said to be intuitively obvious with respect to what structures satisfying the constraint will look like. Using said similarity measures for motif definitions also makes it difficult to strengthen or loosen constraints for specific atoms or atom pairs, while keeping the constraints on all other atom pairs unchanged. A useful alternative to defining motifs through an upper limit on a single-valued similarity measure is to define motifs through distance matrices ** *D* **^U^ and ** *D* **^L^, containing upper and lower bounds, respectively, for some subset *S* of the elements of a distance matrix such as ** *D* **^*A*^ or ** *D* **^*B*^. A configuration of atoms *A* is then said to be an instance of the *n*-atom motif with upper and lower distance limit matrices ** *D* **^U^ and ** *D* **^L^ if and only if:

(5) $$D_{\mathrm{ij}}^{\text{L}}\leq D_{\mathrm{ij}}^{A}\leq D_{\mathrm{ij}}^{\text{U}}$$

for all (*i,j*) ∈ *S*. Defining motifs through upper and lower distance bounds as just described is intuitively straightforward and flexible with respect to which distances to constrain and what constrains to impose on each such distance. For collections of amino acids that are sufficiently small to reappear in multiple unrelated protein structures (*i.e.*, ⪅ 10 aa), it is feasible in practice to search for motifs defined through sets of distance constraints despite the large number of potential combinations implied by Eqn. (2), in part because the set *S* of constrained distances is typically rather small, and in part because candidate configurations may be rejected upon detection of the first constraint violation. For the reasons mentioned, sets of distance constraints are used to specify the geometric aspects of motifs in Swiss-PdbViewer.

**Figure 1:**
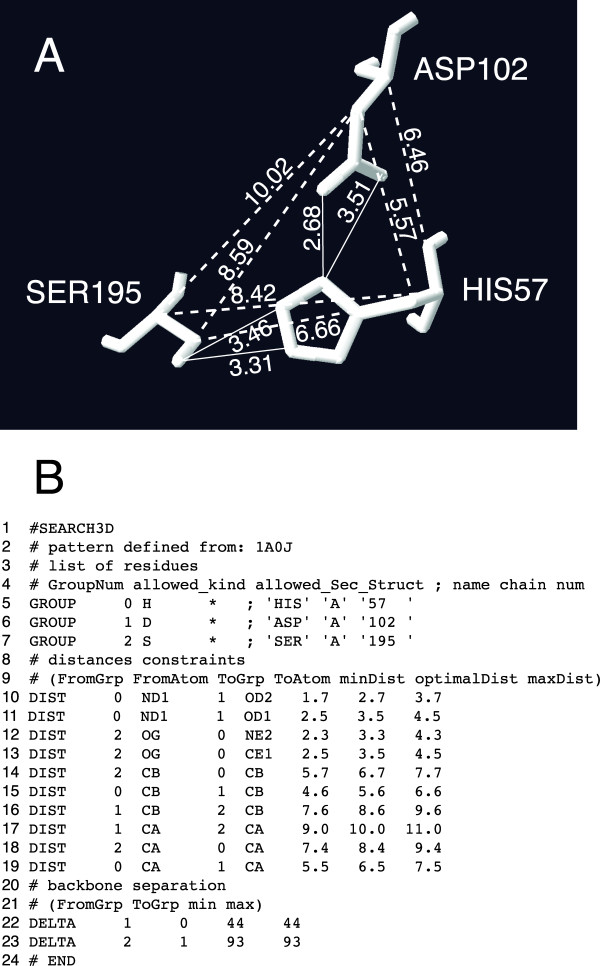
**Motif definition and specification.A)** Structural motif involving residues His57, Asp102 and Ser195 of 1a0j, and interactively measured distances, as rendered by Swiss-PdbViewer (with legibility enhancements). **B)** Textual representation of the motif specification corresponding to the image in Figure 1 (the secondary structure constraints have subsequently been set to “*” using a text editor).

As can be seen in Figure 1B, motif specifications for Swiss-PdbViewer express combinations of amino-acid-type constraints, secondary structure constraints, geometric constraints, and sequence-separation constraints. Combinations of such constraints provide considerable flexibility and are well suited to the specification of partially known, small and sequentially non-consecutive motifs. The described motif specifications and associated search-machinery, are however not intended for, and not well suited to searching for large motifs, such as protein domains or complete proteins.

### DeepView/Swiss-PdbViewer

The program Swiss-PdbViewer (a.k.a. DeepView) [26] was designed to integrate functions for protein structure visualization, analysis and manipulation into a sequence-to-structure workbench with a user-friendly interface. It allows the user to manage complex modelling projects, and Swiss-PdbViewer has been augmented with facilities whereby general structural motifs may be defined and subsequently searched for in a collection of structures (through a web server at the Vital-IT Center for High-Performance Computing of the Swiss Institute of Bioinformatics).

As one example of how to use Swiss-PdbViewer for motif searches, we use the His/Asp/Ser catalytic triad of trypsin from Atlantic salmon (pdb: 1a0j, 1.7 Å resolution). To search for a structural motif such as that represented by His57/Asp102/Ser195, an appropriate set of constraints must be specified. This can be done interactively from within Swiss-PdbViewer by measuring a freely chosen collection of distances (after having opened a pdb structure file, distance measurement mode is activated by clicking on the icon labelled “1.5 Å”. Individual distances are measured by picking pairs of atoms in the structure display window, which is displayed when opening a pdb-file. Distance measurement mode is exited by pressing the keyboard's escape key), as illustrated in Figure 1A, and subsequently selecting the item “Generate 3D Motif from Current Selection …” in the “Tools” menu. Alternatively, programs external to Swiss-PdbViewer can be used to generate motif specifications that can subsequently be opened into Swiss-PdbViewer and used in 3D motif searches as described below. One such program external to Swiss-PdbViewer (the perl-script make-spdbv-motif) is provided in Additional file 1. Since both methods described create motif specifications from existing structures, it is guaranteed that at least one structure satisfying the specification exists. Finally, regardless of which of the two different methods for defining motifs that is used, motif specifications may at present comprise a maximum of 32 groups/residues and up to 150 distance constraints, with a maximum of 31 distance constraints between each pair of residues. The mentioned limits on groups/residues and distance constraints in motif specifications do not represent inherent limitations of the method or its implementation. The limits may be increased in future releases of Swiss-PdbViewer.

A sample motif specification, corresponding to the His/Asp/Ser structural motif, is shown in Figure 1B. As can be seen, motif specifications consist of three parts, each dealing with particular and distinct aspects of the motif, and given by lines of text starting with one of three characteristic keywords (GROUP, DIST or DELTA). In the first part of a motif specification (lines 5–7 in Figure 1B) each residue in the motif is uniquely associated with a numeric group label, followed by residue type and secondary structure restrictions (one of h, s, c, *, hs, hc, or sc, with * meaning no restriction) that need to be satisfied by corresponding residues in actual structures. The alphabetic characters used to specify secondary structure restrictions have the following meanings: h = helix, s = strand, c = coil, and sequences of such characters as well as sequences of single character residue-type abbreviations are seen as being implicitly separated by logical disjunctions. In the second part of a motif specification (lines 10–19 in Figure 1B) distance constraints in Ångström are given that need to hold between specific atoms of the motif residues. The atoms involved in distance constraints are identified by a group label (as given in the first section) and pdb-format atom names, and this is followed by three numeric values, corresponding to the least, measured and greatest distance, respectively. When motif specifications are defined interactively, as described in the previous paragraph, users are prompted to enter a tolerance value (*x*), and the greatest and least value of each distance constraint is set to the distance measured ±*x* Å, (or ± *x*%) respectively. However, all aspects of a motif specification may be further altered using a conventional text editor. In the third part of a motif specification (lines 22–23 in Figure 1B) sequence separation constraints can be given for the residue labels given in the first part of the motif specification. In each sequence-separation constraint, column two and three specify the group labels of the groups between which the constraint shall hold, and columns four and five contain the minimum and maximum sequence separation between the groups in question. Sequence separation constraints are present in motif specifications because it is often desirable to impose restrictions of this kind, but doing so is not a requirement. To avoid imposing sequence separation constraints, corresponding upper limits can be set arbitrarily high (and lower limits set to zero), or the line of text specifying the constraint in question can be left out of the motif specification altogether.

Given a motif specification, individual pdb files as well as a collection of pdb files can be searched for constellations of atoms and amino acid residues that satisfy the constraints in the motif specification. Both of these alternatives are available from within Swiss-PdbViewer, by selecting the item “Search 3D Motif in Current Layer…” or the item “Submit 3D Motif Search Against Subset of PDB…”, both of which are located in the “Tools” menu. The collection of PDB structures currently searched when selecting the second item is the set of 13180 90% non-redundant X-ray structures first mentioned in the section “Common structural motifs in related proteins” above, and in forthcoming releases of Swiss-PdbViewer further PDB-subsets to search will be provided. Submitting a search against a subset of the PDB typically yields a list of hits, for which the constraints of the motif specification used were satisfied.

Upon completion of a search, one line of text is displayed for each combination of residues found to satisfy the constraints of the motif specification used. By clicking on a result line corresponding to a search hit, the appropriate pdb file is loaded into Swiss-PdbViewer and by performing a “Search in Current Layer”, the corresponding residues are selected. It is then easy to superpose the loaded structures and display only the selected residues. The selected residues of each structure are superposed by selecting the item “Fit molecules (from selection)”, located in the “Fit” menu. Through a dialog box, the user is then given the choice of superposing “Carbon Alpha Only”, “Backbone Atoms Only”, “Sidechain Atoms Only” or “All Atoms”, and to select the reference structure onto which the others will be superposed as well as which other structures that are to be superposed onto the reference structure. If not already displayed, an alignment of the amino-acid sequences of loaded structures, with selected residues highlighted, is displayed by selecting the item “Alignment” located in the “Wind” menu. For the purpose of defining and searching for structural motifs, Swiss-PdbViewer is thus a flexible tool, with which the inspection and evaluation of search-results is made easier since sets of residues satisfying structural motifs are kept track of and highlighted in various contexts. In addition, it is of course also possible to analyze or manipulate selected structures and/or substructures using the battery of other tools available for this purpose in Swiss-PdbViewer.

Searching for 3–6-residue motifs in a database of 13180 structures (*vide infra*) takes 80–100 seconds of wall-clock time on a single 2.8 GHz Intel Xeon type processor. The by far most costly part of searches is the reading of files containing molecular structures, and the variations in measured execution times appear not to be correlated with motif size, but instead most likely caused by variations in I/O throughput.

## Results

### Common structural motifs in related proteins

As a first example of a structural motif, we consider the well-known His/Asp/Ser catalytic triad of trypsin (Figure 1A). Using the coordinates of 1a0j, a motif specification such as that shown in Figure 1B, was created interactively using Swiss-PdbViewer. A search for the generated motif specification was performed across a collection of pdb structures (13180 90% non-redundant X-ray structures having a resolution of 3.0 Å or better obtained by using the PISCES sequence culling server [38,39]). A total of 33 sets of atom coordinates satisfying the motif-specification were found, located in 25 uniquely named structures. This corresponds to all structures (and all catalytic triads) present in our database that are related to 1a0j, in the sense of having a blast [40] expectation value (pre-calculated E-value tables were downloaded from rcsb.org) less than 10.0 (the maximum blast E-value observed among these was 1.08·10^-25^). When the search is repeated with all distance constraint error tolerances increased from 1.0 Å to 2.0 Å, the same 33 sets of atom coordinates from 25 uniquely named pdb structures were obtained, indicating that the identified geometric configuration is indeed present as a distinct motif in the corresponding structures.

### Common structural motifs in unrelated proteins

**Figure 2:**
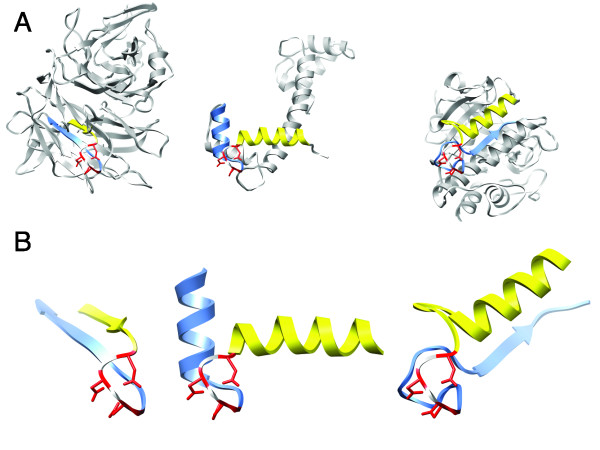
**DxDxDG structural motifs in a variety of structural contexts.A)** Structures of two unrelated hits with pdb ids 1txv (chain **A**, left), 2z30 (chain **A**, right) and 1exr (centre), aligned with respect to their common structural motif. **B)** Expansions of the corresponding structures in **A**, to clearly show the common structural motif. The backbone rmsd:s of the presented structural motifs to the original configuration in 1exr are 0.33 and 0.26 **Å** for 1txv and 2z30, respectively. In both **A)** and **B)**, the residues of each protein that satisfied the motif specification have been coloured red, and the stretches of residues preceding and following the motif have been coloured in yellow and blue, respectively.

As a second example of a structural motif, we consider the well-known, so-called DxDxDG calcium-binding motif of calmodulin [19] that are present in the Ca^2+^-binding helix-loop-helix structures of many calcium-binding proteins. Calmodulin has four calcium-binding motifs (at Asp20, Asp22, Asp24, Gly25, at Asp56, Asp58, Asn60, Gly61, at Asp93, Asp95, Asn97, Gly98, and at Asp129, Asp131, Asp133, Gly134, respectively, in pdb id 1exr), the pairwise backbone rmsd-values between which are all less than 0.5 Å. The second and the third motifs are called DxDxDG-*like* motifs since they have an asparagine in the third position, serine is also common in this position [19]. Using the coordinates of *Paramecium tetraurelia* calmodulin (pdb: 1exr, 1.0 Å resolution), a motif specification (Additional file 2) was created (using the script make-spdbv-motif, in Additional file 1) for the first calcium binding motif given above. Searching for this motif in our non-redundant database returned 134 structural hits located in 54 distinct structures. Of these 54 structures, the primary sequences of 43 have blast E-values of less than 10.0 when compared to the primary sequence of 1exr (the most distant was human frequenin with an E-value of 1.2), and all the DxDxDG EF-hands were retrieved, except two of the four DxDxDG EF-hands present in 2ix7. Quite notably, 2ix7 is an apo-calmodulin bound to the first two IQ motifs of myosin V [41] and the two EF-hands that do not satisfy the distance constraints defined in the motif specification adopt conformations that are clearly distinct from those from which the motif specification was generated. The pdb ids of the remaining 11 structures, considered to be unrelated to 1exr (E-value > 10.0), are 1acc, 1k1x, 1txv, 1ux6, 1wza, 1yo8, 2 h61, 2hpk, 2scp, 2z30 and 2z8r (1acc [anthrax protective antigen], 1k1x [4-alpha-glucanotransferase, C-terminal domain], 1txv [integrin alpha N-terminal domain], 1ux6 [thrombospondin-1], 1wza [bacterial alpha-amylase], 1yo8 [thrombospondin C-terminal domain], 2 h61 [calcyclin (S100)], 2hpk [photoprotein berovin], 2scp [sarcoplasmic calcium-binding protein], 2z30 [tk-subtilisin], 2z8r [YesW protein]). Of these structures, all have a Ca^2+^ ion in the proximity of the three asparagines except 2hpk, in which the Ca^2+^ ion is substituted by a Mg^2+^ ion. Furthermore, it is only in 2hpk, 2 h61 and 2scp that the DxDxDG motifs are situated in the characteristic helix-loop-helix setting, but as has been previously noted the DxDxDG structural motif does occur in a variety of structural contexts [19]. Figures 2A & B show a subset of the mentioned structures aligned with respect to their common DxDxDG motif, and as can be seen, the structures are clearly different aside from their common structural motif.

Our third example of structural motifs, concerns pig insulin. Functional residues are evolutionarily conserved in proteins, and we assumed that residues that contribute to the folding and/or stability of a protein region are also good candidates for conservation. As a part of investigations with a different purpose, residues of importance for the stability of the pig insulin fold have been identified using CMEPS [42] and FOLDEF [43]. According to these results, LeuB11 and LeuB15 (pdb id: 4ins, chain B) are among the most important for fold stability, with the structural neighbors TyrB26 and ValB12 being identified as potential contributor and non-contributor to fold stability, respectively. The importance of ValB12 is on the other hand suggested by experimental results [44].

A motif specification was designed based on LeuB11, ValB12, LeuB15 and TyrB26 in pig insulin (pdb: 4ins, 1.5 Å resolution), with the strict 11-residue sequence separation constraint between the 3^rd^ and 4^th^ residue of the motif loosed by permitting deviations of ±25 (Additional file 3, line 22). As the result of this search, the very same spatial constellation of Leu, Val, Leu and Tyr was found to be present in the His6 enzyme from *Saccharomyces cerevisiae* ([45], pdb: 2agk).

**Figure 3:**
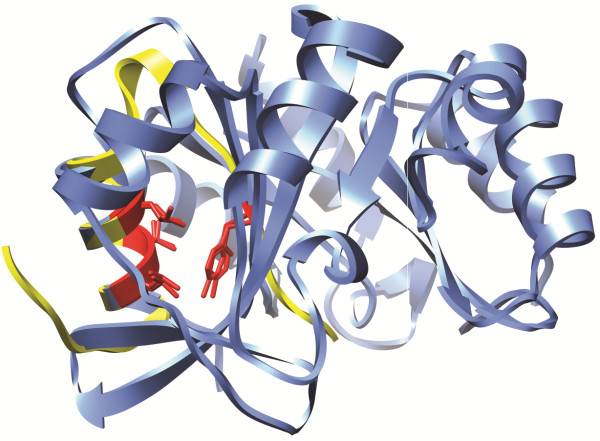
**A deeply buried 4-residue structural motif.** Structures of 4ins chain **B** (yellow) and 2agk (blue), oriented such that the four residues forming the common structural motif (in red) are optimally superposed (the backbone rmsd for the common motif is 0.36 **Å**).

As is clearly seen in Figure 3, the geometries of 4ins and 2agk are very different, with the exception of the four residues forming the motif that was searched for. Although this is *per se* not a proof that these residues contribute to the stability of the yeast protein, the mentioned residues are strictly conserved in homologues of the yeast protein, despite a sequence identity of only about 55% for these homologues. When insulin and insulin-like proteins found in UniProtKB (E < 0.1) are aligned (and assuming Tyr and Phe to be interchangeable at the fourth position), there is a strict sequence conservation of the motif under discussion, except for three sequences (Additional file 4). Two of these sequences originate from *Microcavia niata* and *Galea musteloides*, belonging to the caviomorph group of rodents known to have a deficient insulin that exhibits only 1 – 10% of biological activity in comparison to other mammals [46]. The third sequence comes from the whole genome shotgun of *Tetraodon nigroviridis*, and it would be interesting to have the sequence confirmed and stability checked for this insulin.

When the CMEPS- and FOLDEF-calculations mentioned above are repeated for pdb id 2agk, the residues Leu193, Leu197 and Tyr211 (corresponding to LeuB11, LeuB15 and TyrB26, respectively, in 4ins) were identified as the most important for fold stability and Val194 was identified as a potential contributor to fold stability (Additional file 5).

**Figure 4:**
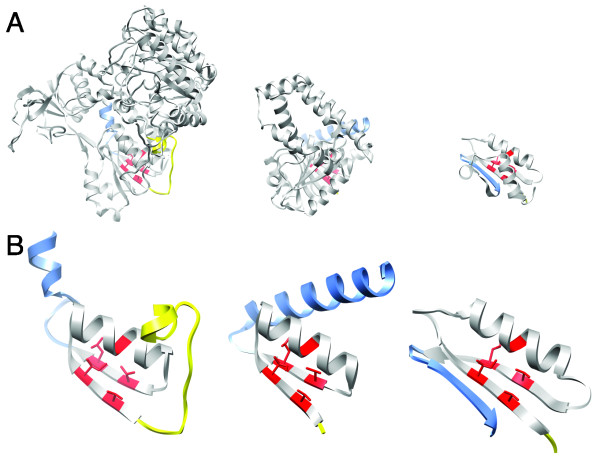
**A deeply buried 5-residue structural motif.A)** Structures of two unrelated hits with pdb ids 1o94 (chain A, left), 2obk (chain A, right) and 2dpo (centre), aligned with respect to their common structural motif. **B)** Expansions of the corresponding structures in **A**, to clearly show the common structural motif. The backbone rmsd:s of the presented structural motifs to the original configuration in 2dpo are 0.38 (1o94) and 0.38 **Å** (2obk), respectively. The structures have been coloured as described in the text for Figure 2.

Our fourth (and final) example of structural motifs concerns rabbit L-gulonate 3-dehydrogenase (pdb: 2dpo, 1.70 Å resolution). Residues Val9, Ile11, Ala22, Val32 and Leu34 in 2dpo are part of the constellation of two β-sheets and an α-helix shown in Figure 4 A & B (centre images). Using the coordinates of 2dpo, a motif specification comprising the above mentioned residues was created (Additional file 6). The defined motif was found to be present (among others) also in pdb ids 1o94 (trimethylamine dehydrogenase) and 2obk (putative Se binding protein), both of which are unrelated to 2dpo (E-value > 10.0). Figures 4 A & B show the mentioned structures aligned with respect to their common motif, and as can be seen, the structures are once again clearly different (ignoring their common structural motif). According to FoldX (Version 2.5.1) [43,47] results, all residues belonging to the above mentioned motifs are expected to be important for the stability of the protein folds, with folding free energy changes upon alanine mutation ranging from 1.8 to 4.3 kcal/mol (Additional file 7, Additional file 8 and Additional file 9, for completeness and comparison, FoldX results for 4ins and 2agk are provided in Additional file 10 and Additional file 11).

## Discussion

The purpose of the examples given above is to illustrate some of the possibilities available when searching for structural motifs using Swiss-PdbViewer. Neither example is intended to be a comprehensive treatment of the corresponding topic (*e.g.*, searching for calcium-binding motifs). Furthermore, searching for structural motifs should not in itself be expected to be competitive with methods developed for and dedicated to identifying specific properties of proteins, such as being calcium-binding [48,49], zinc-binding [50,51], exhibiting catalytic activity [52], *etc.* In particular, methods dedicated to identifying specific properties of proteins are often based on machine-learning techniques and make extensive use of sets of parameters chosen and parameter-values tuned for the specific problem being addressed (*e.g.*, [50,51]).

The examples given above also show that searches for structural motifs can be set up in different ways. Choices of atom-type pairs between which to impose distance constraints, distance constraint tolerance limits, *etc.*, can obviously depend both on what is being searched for and the quality of the structures that are searched. Since motif specifications are text files, they can easily be edited (using conventional text editors) to fit particular user requirements prior to starting/submitting the search. For example, the strict requirement of having an aspartate for the third residue of the motif presented in Additional file 1 could be relaxed to also tolerating an asparagine simply by changing the D to DN. Likewise, individual distance constraints can be tightened or relaxed at will.

As is clearly demonstrated by the examples we have presented, common structural motifs are indeed present and possible to find in evolutionarily and structurally unrelated protein structures in the Protein Data Bank [53]. For the observed motifs, backbone rmsd-values are less than 0.5 Å, which is less than that typically observed across the ensemble for atoms in protein structures determined by NMR spectroscopy [54,55]. Thus, considering the similar geometric configurations of amino acid residues that we have observed in different structures to be instances of common motifs, is well justified.

Previous studies of sequentially non-contiguous structural motifs have been almost exclusively concerned with functional groups on the surfaces of proteins. By contrast, we have also observed structural motifs that exist deeply buried in the interiors of structures (third and fourth example above).

Considering the relative ease with which the given examples were found, we expect such motifs to be a frequently occurring phenomenon. A large number of unanswered questions remain, however. For example, how many such motifs are present on average in each protein structure? In how many distinct structures is a specific motif typically present?, *etc.* Due to the crucial role of side-chain packing in native protein structures, we suspect that structural motifs may become useful for protein structure prediction and refinement.

## Conclusions

Investigations to address the questions posed above, as well as to evaluate the usefulness of structural motifs for structure prediction and refinement are currently underway. Irrespectively, however, it is already clear that the mechanisms to search for structural motifs integrated into DeepView/Swiss-PdbViewer is a useful and valuable tool. The processing time to search for structural motifs of potentially interesting kinds is sufficiently small that it can be used as a standard technique whenever the kinds of information illustrated by our examples would be useful. Furthermore, thanks to being integrated into DeepView/Swiss-PdbViewer, structural motifs can not only be defined by running external programs, but can also be interactively defined with direct visual feedback, from within DeepView/Swiss-PdbViewer. Finally, structure searches, irrespectively of how motifs have been defined, are submitted from within DeepView/Swiss-PdbViewer, so that anyone can benefit from this searching capability without having to maintain a complex hardware/software installation.

## Availability and requirements

Project name: Swiss-PdbViewer

Project home page: http://spdbv.vital-it.ch; tutorial: http://spdbv.vital-it.ch/motif3Dsearch_tut.html

Operating system(s): Microsoft Windows and Mac OS X

Programming language: ANSI C

Other requirements: None

License: freely available in binary/executable form.

Any restrictions to use by non-academics: No

## Competing interests

The authors declare that they have no competing interests.

## Authors’ contributions

NG has designed and implemented SPDBV and conceived the study. MJ performed all analyses, drafted the manuscript and finalized the manuscript with contributions from NG and VZ. VZ performed CMEPS and FoldX calculations. OM contributed to the discussion. All authors read and approved the final manuscript.

## Supplementary Material

Additional file 1 **A perl script (**make-spdbv-motif**) which takes a number of residue id:s (chain id and residue number concatenated into one word) and the name of a pdb-file as arguments and generates a motif specification involving the mentioned residues and the mentioned residues only.** Click here for file

Additional file 2 A DxDxDG motif specification created by the script in Additional file 1. Click here for file

Additional file 3 The motif specification created from pig insulin (pdb id 4ins), with delta-constraints between the second Leu and Tyr loosened by permitting deviations of ±25 from the corresponding sequence separation of the motif in pdb id 4ins. Click here for file

Additional file 4 **Alignment of a fragment of chain B of insulin (4ins) and insulin-like proteins found in UniProtKB. The four residues participating in the structural motif discussed in the text and in Figure**3**are highlighted in bold.** The three sequences which have a residue different than Tyr and Phe at the fourth position are underlined. Click here for file

Additional file 5 The (raw) results of CMEPS calculations of 2agk (see main text for citations) follow immediately below. Bold letters and digits are used for residues and values belonging to the motifs discussed in the text. Energies are in kcal/mol. Click here for file

Additional file 6 The motif specification created from pdb id 2dpo, with distance constraints generated only for distances between Cα atoms, and with delta-constraints between motif-residues Ile and Ala as well as Ala and the last Val loosened by permitting deviations of ±25 from the corresponding sequence separation of the motif in pdb id 2dpo. Click here for file

Additional file 7 The (raw) results of computational alanine scanning of 4ins chain B using FoldX (see main text for citations) follow immediately below. Bold letters and digits are used for residues and values belonging to the motifs discussed in the text. Energies are in kcal/mol. Click here for file

Additional file 8 **The (raw) results of computational alanine scanning of 2agk using FoldX (see main text for citations) follow immediately below.** Bold letters and digits are used for residues and values belonging to the motifs discussed in the text. Energies are in kcal/mol. Click here for file

Additional file 9 **The (raw) results of computational alanine scanning of 2dpo using FoldX (see main text for citations) follow immediately below.** Bold letters and digits are used for residues and values belonging to the motifs discussed in the text. Energies are in kcal/mol. Click here for file

Additional file 10 **The (raw) results of computational alanine scanning of 1o94 using FoldX (see main text for citations) follow immediately below.** Bold letters and digits are used for residues and values belonging to the motifs discussed in the text. Energies are in kcal/mol. Click here for file

Additional file 11 **The (raw) results of computational alanine scanning of 2obk using FoldX (see main text for citations) follow immediately below.** Bold letters and digits are used for residues and values belonging to the motifs discussed in the text. Energies are in kcal/mol. Click here for file
