# Supplementary material for: Defining and searching for structural motifs using DeepView/Swiss-PdbViewer
Source: BMC Bioinformatics. 2012 Jul 23;13:173. doi: 10.1186/1471-2105-13-173 (PMC3436773; doi:10.1186/1471-2105-13-173)
Supplement: Additional file 1 — A perl script (make-spdbv-motif) which takes a number of residue id:s (chain id and residue number concatenated into one word) and the name of a pdb-file as arguments and generates a motif specification involving the mentioned residues and the mentioned residues only. [file 1471-2105-13-173-S3.pdf]

Additonal file 3 The motif specification created from pig insulin (pdb id 4ins), with delta-constraints between the second Leu and Tyr loosened by permitting deviations of ±25 from the corresponding sequence separation of the motif in pdb id 4ins.

```
#SEARCH3D
# pattern defined from: 4INS
# list of residues
# GroupNum allowed_kind allowed_Sec_Struct ; name chain num
GROUP     0 L      h    ; 'LEU' 'B' '11  '
GROUP     1 V      h    ; 'VAL' 'B' '12  '
GROUP     2 L      h    ; 'LEU' 'B' '15  '
GROUP     3 Y      s    ; 'TYR' 'B' '26  '
# distances constraints
# (FromGrp FromAtom ToGrp ToAtom minDist optimalDist maxDist)
DIST     3  CZ       1  CB     4.8    5.8    6.8
DIST     3  CE1      0  CG     3.7    4.7    5.7
DIST     3  CD1      2  CG     4.6    5.6    6.6
DIST     3  CG       1  CB     4.5    5.5    6.5
DIST     0  CG       1  CB     4.9    5.9    6.9
DIST     1  CB       2  CG     5.0    6.0    7.0
DIST     2  CG       0  CG     3.9    4.9    5.9
# backbone separation
# (FromGrp ToGrp min max)
DELTA     1     0     1     1
DELTA     2     1     3     3
DELTA     3     2     1    36
# END
```
