# Supplementary material for: Defining and searching for structural motifs using DeepView/Swiss-PdbViewer
Source: BMC Bioinformatics. 2012 Jul 23;13:173. doi: 10.1186/1471-2105-13-173 (PMC3436773; doi:10.1186/1471-2105-13-173)
Supplement: Additional file 2 — A DxDxDG motif specification created by the script in Additional file 1. [file 1471-2105-13-173-S4.pdf]

Additional file 4 Alignment of a fragment of chain B of insulin (4ins) and insulin-like proteins found in UniProtKB. The four residues participating in the structural motif discussed in the text and in Figure 3 are highlighted in bold. The three sequences which have a residue different than Tyr and Phe at the fourth position are underlined.

column 1 = sequence of fragment
column 2 = accession
column 3 = protein name
column 4 = organism followed by E-value

LVDA**L**Y-FVCGERGFF**Y** P68992 Insulin Hydrolagus colliei (Spotted ratfish) (Pacific ratfish) 2.0×10-12
LVDA**L**Y-FVCGERGFF**Y** P68991 Insulin Chimaera monstrosa (Rabbit fish) 2.0×10-12
LVDA**L**Y-FVCGERGFF**Y** P13190 Insulin Callorhynchus milii (Elephant fish) (Australian ghost shark) 2.0×10-12
LVEA**L**Y-FVCGPKGFY**Y** P12705 Insulin Torpedo marmorata (Marbled electric ray) 6.0×10-11
LVEA**L**Y-FVCGPKGFY**Y** P12704 Insulin Squalus acanthias (Spiny dogfish) 3.0×10-10
LVEA**L**Y-LVCGERGFF**Y** P69048 Insulin Trachemys scripta (Red-eared slider turtle) (Pseudemys scripta) 1.0×10-17
LVEA**L**Y-LVCGERGFF**Y** P67968 Insulin Meleagris gallopavo (Common turkey) 1.0×10-17
LVEA**L**Y-LVCGERGFF**Y** P69047 Insulin Chrysemys dorbigni (Black-bellied slider turtle) (Trachemys dorbigni) 1.0×10-17
LVEA**L**Y-LVCGERGFF**Y** P67969 Insulin Struthio camelus (Ostrich) 1.0×10-17
LVEA**L**Y-LVCGDRGFF**Y** B5AK54 Insulin Xenopus tropicalis (Western clawed frog) (Silurana tropicalis) 2.0×10-15
LVEA**L**Y-LVCGDRGFF**Y** A4IGV9 Ins protein Xenopus tropicalis (Western clawed frog) (Silurana tropicalis) 2.0×10-15
LVEA**L**Y-LVCGDRGFF**Y** B7ZQP1 Insulin-like growth factor 2 (Somatomedin A) Xenopus laevis (African clawed frog) 2.0×10-15
LVEA**L**Y-LVCGDRGFF**Y** P12707 Insulin-2 Xenopus laevis (African clawed frog) 2.0×10-15
LVEA**L**Y-LVCGDRGFF**Y** P12706 Insulin-1 Xenopus laevis (African clawed frog) 3.0×10-16
LVEA**L**F-LVCGDRGFF**Y** Q7LZM9 Insulin Amphiuma tridactylum (three-toed salamander) 2.0×10-14
LVEA**L**Y-MVCGDRGFF**Y** Q9I8Q7 PreproInsulin Rana pipiens (Northern leopard frog) 4.0×10-13
LVDA**L**Y-LVCGERGFF**Y** P12703 Insulin Alligator mississippiensis (American alligator) 9.0×10-15
LVEA**L**Y-LVCGERGFF**Y** P68243 Insulin Cairina moschata (Muscovy duck) 6.0×10-17
LVEA**L**Y-LVCGERGFF**Y** P01333 Insulin Anas platyrhynchos (Domestic duck) (Anas boschas) 6.0×10-17
LVEA**L**Y-LVCGERGFF**Y** P68245 Insulin Anser anser anser (Western graylag goose) 6.0×10-17
LVEA**L**Y-LVCGERGFF**Y** Q7M0U6 Epidermal growth factor/single chain insulin ... Brevibacillus brevis (Bacillus brevis) 7.0×10-20
LVEA**L**Y-LVCGERGFF**Y** P01340 Insulin Katsuwonus pelamis (Skipjack tuna) (Bonito) 6.0×10-15
LVDA**L**Y-MVCGEKGFF**Y** Q90ZY1 PreproInsulin Hiodon alosoides (goldeye) 9.0×10-13
LVDA**L**Y-MVCGEKGFF**Y** Q98TA8 Insulin Pantodon buchholzi (Freshwater butterflyfish) 9.0×10-13
LVEA**L**F-LVCGERGFF**F** Q98TA9 PreproInsulin Gnathonemus petersii (elephantnose fish) 9.0×10-13
LVDA**L**Y-MVCGDRGFF**Y** Q98TA7 PreproInsulin Osteoglossum bicirrhosum (silver arawana) 3.0×10-12
LVEA**L**Y-LVCGERGFF**Y** Q98TB0 PreproInsulin Chitala chitala (clown knifefish) 1.0×10-16
LVDA**L**Y-LVCGEKGLF**Y** B5XDT4 Insulin Salmo salar (Atlantic salmon) 2.0×10-12
LVDA**L**Y-LVCGEKGLF**Y** B5X6U5 Insulin Salmo salar (Atlantic salmon) 2.0×10-12
LVDA**L**Y-LVCGEKGFF**Y** A3RID6 Preproinsulin 1 Oncorhynchus mykiss (Rainbow trout) (Salmo gairdneri) 7.0×10-14
LVDA**L**Y-LVCGEKGFF**Y** P04667 Insulin Oncorhynchus keta (Chum salmon) 5.0×10-15
LVDA**L**Y-LVCGEKGFF**Y** B5XEB7 Insulin Salmo salar (Atlantic salmon) 7.0×10-14
LVDA**L**Y-LVCGEKGFF**Y** A3RID7 Preproinsulin 2 Oncorhynchus mykiss (Rainbow trout) (Salmo gairdneri) 7.0×10-14
LVDA**L**Y-LVCGEKGFF**Y** P68990 Insulin Oncorhynchus kisutch (Coho salmon) 7.0×10-14
LVDA**L**Y-LVCGEKGFF**Y** P68989 Insulin Oncorhynchus gorbuscha (Pink salmon) (Humpback salmon) 7.0×10-14
LVDA**L**Y-LVCGPTGFF**Y** A5PF52 PreproInsulin Danio rerio (Zebrafish) (Brachydanio rerio) 2.0×10-11
LVDA**L**Y-LVCGPTGFF**Y** O73727 Insulin Danio rerio (Zebrafish) (Brachydanio rerio) 2.0×10-11
LVDA**L**Y-LVCGPTGFF**Y** Q9DDE5 Insulin Danio rerio (Zebrafish) (Brachydanio rerio) 2.0×10-11
LVDA**L**Y-LVCGPTGFF**Y** Q90ZN4 PreproInsulin Catla catla (catla) 2.0×10-11
LVDA**L**Y-LVCGPTGFF**Y** P01335 Insulin Cyprinus carpio (Common carp) 2.0×10-11
LVDA**L**Y-LVCGPTGFF**Y** Q98TB1 PreproInsulin Catostomus commersonii (White sucker) 2.0×10-11
LVDA**L**Y-LVCGPSGFF**Y** P81881 Insulin Piaractus mesopotamicus (Pacu) 7.0×10-12
LVDA**L**Y-LVCGDRGFF**Y** Q98TB2 PreproInsulin Ambloplites rupestris (Rock bass) 4.0×10-14
LVDA**L**Y-LVCGDRGFF**Y** Q18NR0 Insulin Seriola dumerili (Mediterranean greater amberjack) (Caranx dumerili) 4.0×10-14
LVDA**L**Y-LVCGDRGFF**Y** P69045 Insulin Lophius americanus (American goosefish) (Anglerfish) 4.0×10-14
LVDA**L**Y-LVCGERGFF**Y** Q9W7R2 Insulin Verasper moseri (Barfin flounder) 3.0×10-15
LVDA**L**Y-LVCGDRGFF**Y** P81025 Insulin Oreochromis niloticus (Nile tilapia) (Tilapia nilotica) 3.0×10-13
LVDA**L**Y-LVCGDRGFF**Y** Q4SPT8 Chromosome 7 SCAF14536, whole genome shotgun ... Tetraodon nigroviridis (Green puffer) 4.0×10-14
LVDA**L**Y-LVCGDRGFF**Y** P01339 Insulin Thunnus thynnus (Atlantic bluefin tuna) (Scomber thynnus) 4.0×10-14
LVDA**L**Y-LVCGDRGFF**Y** P69046 Insulin Lophius piscatorius (Allmouth goosefish) (Anglerfish) 4.0×10-14
LVDA**L**Y-LVCGDRGFF**Y** P01336 Insulin Gadus callarias (Baltic cod) 4.0×10-14
LVDA**L**Y-LVCGDRGFF**Y** P01337 Insulin-1 Batrachoididae sp. (Toadfish) 4.0×10-14
LVDA**L**Y-LVCGDRGFF**Y** P01338 Insulin 2 Batrachoididae sp. (Toadfish) 2.0×10-12
LVDA**L**Y-LVCGERGFF**Y** P09477 Insulin Platichthys flesus (European flounder) (Pleuronectes flesus) 3.0×10-15
LVEA**L**Y-LVCGDRGFF**Y** Q05K39 Insulin Oryzias latipes (Medaka fish) (Japanese ricefish) 5.0×10-16
LVDA**L**H-LICGDRGFI**Y** Q18NR1 Insulin Seriola dumerili (Mediterranean greater amberjack) (Caranx dumerili) 9.0×10-8
LVDA**L**Y-LVCGDRGFF**Y** P07453 Insulin Myoxocephalus scorpius (Shorthorn sculpin) (Cottus scorpius) 4.0×10-14
LVDA**L**Y-LVCGPRGFF**Y** Q2QAJ9 Preproinsulin 2 Danio rerio (Zebrafish) (Brachydanio rerio) 1.0×10-10
LVEA**L**Y-LVCGSNGFF**F** P42633 Insulin Anguilla rostrata (American eel) (Muraena rostrata) 2.0×10-11
LVEA**L**Y-LVCGEKGFF**Y** P09476 Insulin Atractosteus spatula (Alligator gar) (Lepisosteus spatula) 9.0×10-15
LVEA**L**F-LVCGESGFF**Y** P29335 Insulin Amia calva (Bowfin) 2.0×10-12
LVEA**L**Y-VVCGDRGFF**Y** P68987 Insulin Petromyzon marinus (Sea lamprey) 5.0×10-13
LVEA**L**Y-VVCGDRGFF**Y** P68988 Insulin Lampetra fluviatilis (European river lamprey) (Petromyzon fluviatilis) 5.0×10-13
LVNA**L**Y-IACGVRGFF**Y** P01342 Insulin Myxine glutinosa (Atlantic hagfish) 3.0×10-5
LVEA**L**Y-LVCGNRGFF**Y** P0C236 Insulin Polypterus senegalus (Senegal bichir) 3.0×10-13
LVEA**L**Y-LVCGERGFF**Y** Q7LZN0 Insulin I1 Polyodon spathula (North American paddlefish) (Squalus spathula) 6.0×10-17
LVEA**L**Y-LVCGERGFF**Y** P81423 Insulin Acipenser guldenstadti (Caspian sturgeon) (Russian sturgeon) 6.0×10-17
LVEA**L**Y-LVCGERGFF**Y** P01310 Insulin Equus caballus (Horse) 1.0×10-20
LVEA**L**Y-LVCGERGFF**Y** P01316 Insulin Elephas maximus (Indian elephant) 7.0×10-20
LVEA**L**Y-LVCGERGFF**Y** Q52PU3 PreproInsulin Felis catus (Cat) (Felis silvestris catus) 1.0×10-20
LVEA**L**Y-LVCGERGFF**Y** P06306 Insulin Felis catus (Cat) (Felis silvestris catus) 1.0×10-20
LVEA**L**Y-LVCGERGFF**Y** Q7M217 Insulin Canavalia ensiformis (Jack bean) (Horse bean) 1.0×10-20
LVEA**L**Y-LVXGERGFF**Y** P83770 Insulin-like protein Vigna unguiculata (Cowpea) 7.0×10-18
LVEA**L**Y-LVCGERGFF**Y** P01320 Insulin Camelus dromedarius (Dromedary) (Arabian camel) 2.0×10-19
LVEA**L**Y-LVCGERGFF**Y** Q25C78 ProInsulin Bubalus bubalis (Domestic water buffalo) 1.0×10-20
LVEA**L**Y-LVCGERGFF**Y** P01317 Insulin Bos taurus (Bovine) 1.0×10-20
LVEA**L**Y-LVCGERGFF**Y** A5PJB2 Ins protein Bos taurus (Bovine) 1.0×10-20
LVEA**L**Y-LVCGERGFF**Y** P01318 Insulin Ovis aries (Sheep) 1.0×10-20
LVEA**L**Y-LVCGERGFF**Y** P01319 Insulin Capra hircus (Goat) 1.0×10-20
LVEA**L**Y-LVCGERGFF**Y** P01314 Insulin Balaenoptera borealis (Sei whale) (Pollack whale) 1.0×10-20
LVEA**L**Y-LVCGERGFF**Y** B5FWC2 Insulin (Predicted) Otolemur garnettii (Small-eared galago) (Garnett's greater bushbaby) 1.0×10-20
LVEA**L**Y-LVCGERGFF**Y** P18109 Insulin Didelphis marsupialis virginiana (North American opossum) 2.0×10-19
LVEA**L**Y-LVCGERGFF**Y** P67974 Insulin Physeter macrocephalus (Sperm whale) (Physeter catodon) 1.0×10-20
LVEA**L**Y-LVCGERGFF**Y** B3RFH3 Insulin (Predicted) Sorex araneus (Eurasian common shrew) (European shrew) 4.0×10-19
LVEA**L**Y-LVCGERGFF**Y** P67973 Insulin Balaenoptera physalus (Finback whale) (Common rorqual) 1.0×10-20

LVEA**L**Y-LVCGERGFF**Y** Q8HZ81 Insulin Gorilla gorilla (Western Gorilla) 9.0×10-14
LVEA**L**Y-LVCGERGFF**Y** Q8HZ80 Insulin Pongo pygmaeus (Bornean orangutan) 9.0×10-14
LVEA**L**Y-LVCGERGFF**Y** P30410 Insulin Pan troglodytes (Chimpanzee) 7.0×10-20
LVEA**L**Y-LVCGERGFF**Y** P01308 Insulin Homo sapiens (Human) 7.0×10-20
LVEA**L**Y-LVCGERGFF**Y** Q6YK33 Insulin Gorilla gorilla gorilla (Lowland gorilla) 7.0×10-20
LVEA**L**Y-LVCGERGFF**Y** Q8HXV2 Insulin Pongo pygmaeus (Bornean orangutan) 7.0×10-20
LVEA**L**Y-LVCGERGFF**Y** P30406 Insulin Macaca fascicularis (Crab-eating macaque) (Cynomolgus monkey) 7.0×10-20
LVEA**L**Y-LVCGERGFF**Y** P30407 Insulin Cercopithecus aethiops (Green monkey) (Grivet) 7.0×10-20
LVEA**L**Y-LVCGERGFF**Y** A6XGL2 Insulin Homo sapiens (Human) 7.0×10-20
LVEA**L**Y-LVCGERGFF**Y** P67971 Insulin Saimiri sciureus (Common squirrel monkey) 7.0×10-18
LVEA**L**Y-LVCGERGFF**Y** P67972 Insulin Aotus trivirgatus (Night monkey) (Douroucouli) 7.0×10-18
LVEA**L**Y-LVCGERGFF**Y** B2KIN7 Proinsulin (Predicted) Rhinolophus ferrumequinum (Greater horseshoe bat) 1.0×10-20
LVEA**L**Y-LVCGERGFF**Y** P01315 Insulin Sus scrofa (Pig) 1.0×10-20
LVEA**L**Y-LVCGERGFF**Y** P01321 Insulin Canis familiaris (Dog) (Canis lupus familiaris) 1.0×10-20
LVEA**L**Y-LVCGERGFF**Y** Q1WM24 InsIGF long transcript variant Homo sapiens (Human) 7.0×10-20
LVEA**L**Y-LVCGERGFF**Y** C9JNR5 Uncharacterized protein Homo sapiens (Human) 7.0×10-20
LVEA**L**Y-LVCGERGFF**Y** D2HDS9 Putative uncharacterized protein Ailuropoda melanoleuca (Giant panda) 1.0×10-20
LVEA**L**Y-LVCGERGFF**Y** D3Z595 Uncharacterized protein Mus musculus (Mouse) 7.0×10-18
LVEA**L**Y-LVCGERGFF**Y** D3YWU5 Uncharacterized protein Mus musculus (Mouse) 7.0×10-18
LVEA**L**Y-LVCGERGFF**Y** D3Z596 Uncharacterized protein Mus musculus (Mouse) 7.0×10-18
LVEA**L**Y-LVCGERGFF**Y** Q6XVL6 PreproInsulin Passer domesticus (House sparrow) (Fringilla domestica) 6.0×10-18
LVEA**L**Y-LVCGERGFF**Y** Q6XVL4 PreproInsulin Pica pica (Black-billed magpie) 6.0×10-17
LVEA**L**Y-LVCGERGFF**Y** Q6XVK7 PreproInsulin Turdus merula (Blackbird) 6.0×10-18
LVEA**L**Y-LVCGERGFF**Y** Q6XVN0 PreproInsulin Aptenodytes forsteri (Emperor penguin) 1.0×10-17
LVEA**L**Y-LVCGERGFF**Y** Q6XVM3 PreproInsulin Fulmarus glacialis (Northern fulmar) 1.0×10-17
LVEA**L**Y-LVCGERGFF**Y** Q6XVL7 PreproInsulin Pagodroma nivea (lesser snow-petrel) 1.0×10-17
LVEA**L**Y-LVCGERGFF**Y** Q6XVM4 PreproInsulin Falco tinnunculus (common kestrel) 1.0×10-16
LVEA**L**Y-LVCGERGFF**Y** Q6XVL9 PreproInsulin Melopsittacus undulatus (Budgerigar) 1.0×10-16
LVEA**L**Y-LVCGERGFF**Y** Q6XVM2 PreproInsulin Gallinula chloropus (Common moorhen) 1.0×10-16
LVEA**L**Y-LVCGERGFF**Y** Q6XVM8 PreproInsulin Ciconia ciconia (White stork) 1.0×10-17
LVEA**L**Y-LVCGERGFF**Y** Q6XVM6 PreproInsulin Thalassarche melanophrys (Black-browed albatross) 1.0×10-17
LVEA**L**Y-LVCGERGFF**Y** Q6XVL8 PreproInsulin Nycticorax nycticorax hoactli 1.0×10-17
LVEA**L**Y-LVCGERGFF**Y** Q6XVL1 PreproInsulin Strix aluco (Tawny owl) 1.0×10-17
LVEA**L**Y-LVCGERGFF**Y** Q6XVK9 PreproInsulin Sula sula (Red-footed booby) 1.0×10-17
LVEA**L**Y-LVCGERGFF**Y** Q6XVL5 PreproInsulin Phaethon aethereus (Red-billed tropicbird) 1.0×10-17
LVEA**L**Y-LVCGERGFF**Y** Q6XVL2 PreproInsulin Rissa tridactyla (Black-legged kittiwake) 1.0×10-17
LVEA**L**Y-LVCGERGFF**Y** Q6XVK6 PreproInsulin Uria aalge (Common guillemot) (Common murre) 1.0×10-17
LVEA**L**Y-LVCGERGFF**Y** Q6XVL0 PreproInsulin Struthio camelus (Ostrich) 1.0×10-17
LVEA**L**Y-LVCRERGFF**Y** Q6XVL3 PreproInsulin Rhea americana (Greater rhea) (Common rhea) 8.0×10-16
LVEA**L**Y-LVCGERGFF**Y** Q6XVN3 PreproInsulin Anas crecca (Green-winged teal) 6.0×10-17
LVEA**L**Y-LVCGERGFF**Y** Q6XVN2 PreproInsulin Anas platyrhynchos (Domestic duck) (Anas boschas) 6.0×10-17
LVEA**L**Y-LVCGERGFF**Y** Q6XVM9 PreproInsulin Cairina moschata (Muscovy duck) 6.0×10-17
LVEA**L**Y-LVCGERGFF**Y** Q6XVK8 PreproInsulin Tadorna tadorna (common shelduck) 6.0×10-17
LVEA**L**Y-LVCGERGFF**Y** Q6XVM5 PreproInsulin Dromaius novaehollandiae (Emu) 1.0×10-17
LVEA**L**Y-LVCGERGFF**Y** Q6XVM7 PreproInsulin Columba livia (Domestic pigeon) 1.0×10-17
LVEA**L**Y-LVCGERGFF**Y** Q6XVM1 PreproInsulin Gallus gallus (Chicken) 1.0×10-17
LVEA**L**Y-LVCGERGFF**Y** Q6XVM0 PreproInsulin Meleagris gallopavo (Common turkey) 1.0×10-17
LVEA**L**Y-LVCGERGFF**Y** P67970 Insulin Gallus gallus (Chicken) 1.0×10-17
LVEA**L**Y-LVCGERGFF**Y** P51463 Insulin Selasphorus rufus (Rufous hummingbird) 1.0×10-18
LVEA**L**YILVCGERGFF**Y** P21563 Insulin Rodentia sp. 1.0×10-17
LVEA**L**Y-LVCGERGFF**Y** A0ELZ0 PreproInsulin Meriones unguiculatus (Mongolian jird) (Mongolian gerbil) 7.0×10-20
LVEA**L**Y-LVCGERGFF**Y** Q62587 Insulin Psammomys obesus (Fat sand rat) 7.0×10-20
LVEA**L**Y-LVCGERGFF**Y** A0ELZ1 PreproInsulin Volemys kikuchii (Taiwanese vole) (Microtus kikuchii) 7.0×10-20
LVEA**L**Y-LVCGERGFF**Y** P01313 Insulin Cricetulus longicaudatus (Long-tailed dwarf hamster) (Chinese hamster) 7.0×10-20
LVEA**L**Y-LVCGERGFF**Y** Q5EEX1 Insulin II Mus musculus (Mouse) 7.0×10-18
LVEA**L**Y-LVCGERGFF**Y** D0EY27 Insulin-2 Mus musculus (Mouse) 7.0×10-18
LVEA**L**Y-LVCGERGFF**Y** Q2IDG8 Insulin II Mus spretus (Western Mediterranean mouse) (Algerian mouse) 7.0×10-18
LVEA**L**Y-LVCGERGFF**Y** P01326 Insulin-2 Mus musculus (Mouse) 7.0×10-18
LVEA**L**Y-LVCGERGFF**Y** A0ELY8 Preproinsulin 2 Mus caroli (Ryukyu mouse) (Ricefield mouse) 7.0×10-18
LVEA**L**Y-LVCGERGFF**Y** E0CXX7 Uncharacterized protein Mus musculus (Mouse) 7.0×10-18
LVEA**L**Y-LVCGERGFF**Y** A0ELY6 Preproinsulin 2 Rattus losea (lesser rice-field rat) 7.0×10-18
LVEA**L**Y-LVCGERGFF**Y** P01323 Insulin-2 Rattus norvegicus (Rat) 7.0×10-18
LVEA**L**Y-LVCGERGFF**Y** A0ELY9 Preproinsulin 2 Niviventer coxingi (Coxing's white-bellied rat) 7.0×10-18
LVEA**L**Y-LVCGERGFF**Y** A0ELY7 Preproinsulin 2 Apodemus semotus (Taiwan field mouse) (Formosan wood mouse) 7.0×10-18
LVEA**L**Y-LVCGERGFF**Y** A0ELY2 Preproinsulin 1 Rattus losea (lesser rice-field rat) 1.0×10-17
LVEA**L**Y-LVCGERGFF**Y** P01322 Insulin-1 Rattus norvegicus (Rat) 1.0×10-17
LVEA**L**Y-LVCGERGFF**Y** A0ELY5 Preproinsulin 1 Niviventer coxingi (Coxing's white-bellied rat) 1.0×10-17
LVEA**L**Y-LVCGERGFF**Y** Q545I7 Insulin 1 Mus musculus (Mouse) 1.0×10-17
LVEA**L**Y-LVCGERGFF**Y** P01325 Insulin-1 Mus musculus (Mouse) 1.0×10-17
LVEA**L**Y-LVCGERGFF**Y** A0ELY4 Preproinsulin 1 Mus caroli (Ryukyu mouse) (Ricefield mouse) 8.0×10-17
LVEA**L**Y-LVCGERGFF**Y** A0ELY3 Preproinsulin 1 Apodemus semotus (Taiwan field mouse) (Formosan wood mouse) 1.0×10-17
LVEA**L**Y-LVCGERGFF**Y** P01324 Insulin Acomys cahirinus (Egyptian spiny mouse) 1.0×10-19
LVEA**L**Y-LVCGERGFF**Y** Q7M0G1 Insulin Cricetidae sp. (Hamster) 7.0×10-20
LVEA**L**Y-LVCGERGFF**Y** B7NZU4 Insulin (Predicted) Oryctolagus cuniculus (Rabbit) 7.0×10-20
LVEA**L**Y-LVCGERGFF**Y** P01311 Insulin Oryctolagus cuniculus (Rabbit) 7.0×10-20
LVEA**L**Y-LVCGERGFF**Y** Q91XI3 Insulin Spermophilus tridecemlineatus (Thirteen-lined ground squirrel) (Ictidomys tridecemlineatus) 7.0×10-20
LVDA**L**Y-LVCGDRGFF**Y** Q5BVF6 Insulin Chinchilla chinchilla (Short-tailed chinchilla) (Chinchilla brevicaudata) 1.0×10-16
LVDA**L**Y-LVCGDRGFF**Y** P01327 Insulin Chinchilla chinchilla (Short-tailed chinchilla) (Chinchilla brevicaudata) 1.0×10-16
LVDA**L**Y-LVCGDRGFF**Y** Q5BVF5 Insulin Chinchilla lanigera (Long-tailed chinchilla) (Chinchilla villidera) 1.0×10-16
LVEA**L**Y-MVCRDKGFF**Y** Q5BVF4 Insulin Lagidium viscacia (southern viscacha) 6.0×10-12
LVDA**L**Y-SACGDRGFF**Y** Q5BVE9 Insulin Dolichotis patagonum (Patagonian cavy) 7.0×10-13
LVDA**L**Y-SACGDKGFF**Y** Q5BVE8 Insulin Hydrochoerus hydrochaeris (Capybara) (Carpincho) 4.0×10-11
LVET**L**Y-SVCQDDGFF**Y** Q5BVF3 Insulin Cavia porcellus (Guinea pig) 9.0×10-8
LVET**L**Y-SVCQDDGFF**Y** Q5BVF2 Insulin Cavia aperea (Brazilian guinea pig) 9.0×10-8
LVET**L**Y-SVCQDDGFF**Y** P01329 Insulin Cavia porcellus (Guinea pig) 9.0×10-8
LVET**L**Y-EVCRDKGFF**S** Q5BVF1 Insulin Microcavia niata 2.0×10-7
LVDA**L**Y-VVCKDKGFF**S** Q5BVF0 Insulin Galea musteloides (Common yellow-toothed cavy) 1.0×10-6
LVEA**L**Y-MACGDKGFF**Y** Q5BVE7 Insulin Dasyprocta punctata (punctate agouti) 3.0×10-16
LVEA**L**Y-LVCGNDGFF**Y** P01328 Insulin Hystrix cristata (North African crested porcupine) 2.0×10-15
LVEA**L**Y-LVCGEKGFY**Y** Q9TQY7 Insulin Ornithorhynchus anatinus (Duckbill platypus) 3.0×10-14
LVEA**L**F-LICGERGFY**Y** P12708 Insulin Zaocys dhumnades (Big-eyed ratsnake) (Ptyas dhumnades) 4.0×10-12
LVEA**L**F-LICGERGFY**Y** P01334 Insulin Crotalus atrox (Western diamondback rattlesnake) 4.0×10-12
LVEA**L**Y-MTCGHNG-F**Y** Q5BVE0 Insulin Ctenomys leucodon (White-toothed tuco-tuco) 2.0×10-6
LVEA**L**Y-MTCGHNG-F**Y** Q5BVD9 Insulin Ctenomys rionegrensis 2.0×10-6
LVEA**L**Y-MTCGHNG-F**Y** Q5BVD8 Insulin Ctenomys robore 2.0×10-6
LVEA**L**Y-MTCGHSG-F**Y** Q5BVE6 Insulin Tympanoctomys barrerae (Plains viscacha rat) 1.0×10-5
LVEA**L**Y-MTCGRSG-F**Y** Q5BVE3 Insulin Spalacopus cyanus (Coruro) 2.0×10-5
LVEA**L**Y-MTCGHSG-F**Y** Q5BVE5 Insulin Octomys mimax (viscacha rat) 1.0×10-5
LVEA**L**Y-MTCGHSG-F**Y** Q5BVE4 Insulin Aconaemys fuscus 1.0×10-5
LVEA**L**Y-MTCGHNG-F**Y** Q5BVE1 Insulin Octodontomys gliroides (Mountain degu) 3.0×10-5

LVEALY-MTCGRSG-FY Q5BVE2 Insulin Octodon degus (Degu) (Sciurus degus) 2.0×10-5
LVEALY-MTCGRSG-FY P17715 Insulin Octodon degus (Degu) (Sciurus degus) 2.0×10-5
LVDTLY-SVCRHRG-FY P01330 Insulin Myocastor coypus (Coypu) (Nutria) 2.0×10-3
LVDTLY-SVCRHRG-FY Q5BVD7 Insulin Hoplomys gymnurus (Armored rat) (Echimys gymnurus) 5.0×10-3
LVDTLY-SVCKHRG-FY P01331 Insulin Proechimys guairae (Guaira spiny rat) 3.0×10-3
LVDALF-LLCERNG-FY Q5BVD6 Insulin Abrocoma bennettii (Bennett's chinchilla rat) 6.0×10-3
LVDTLY-FICGERGFYA Q4T1L5 Chromosome 1 SCAF10546, whole genome shotgun ... Tetraodon nigroviridis (Green puffer) 3.0×10-7
LVDALQ-FVCGERGFYF Q8SQC4 Insulin-like growth factor 1 Trichosurus vulpecula (Brush-tailed possum) 1.6×10-2
LVDALQ-FVCGERGFYF D7PCU0 Insulin-like growth factor 1 Trichosurus vulpecula (Brush-tailed possum) 1.6×10-2
LVDALQ-FVCGERGFYF B7T506 Insulin-like growth factor-1 Macropus eugenii (Tammar wallaby) 1.6×10-2
LVDALQ-FVCGDRGFYF A2IRL3 Insulin like growth factor I Cynops pyrrhogaster (Japanese common newt) 3.8×10-2
LVDALQ-FVCGDRGFYF Q14WA7 Insulin-like growth factor I Anser anser (domestic goose) 9.1×10-2
LVDALQ-FVCGDRGFYF A2IT63 Insulin-like growth factor-I Anas platyrhynchos (Domestic duck) (Anas boschas) 9.1×10-2
LVDALQ-FVCGDRGFYF A7LKM7 Insulin-like growth factor 1 Anas platyrhynchos (Domestic duck) (Anas boschas) 9.1×10-2
LVDALQ-FVCGDRGFYF Q1G348 Insulin-like growth factor-1 Anser anser (domestic goose) 9.1×10-2
LVDALQ-FVCGDRGFYF C5J073 Insulin-like growth factor Gallus gallus (Chicken) 9.1×10-2
LVDALQ-FVCGDRGFYF P18254 Insulin-like growth factor I Gallus gallus (Chicken) 9.1×10-2
LVDALQ-FVCGDRGFYF P51462 Insulin-like growth factor I Coturnix coturnix japonica (Japanese quail) (Coturnix japonica) 9.1×10-2
LVDALQ-FVCGDRGFYF O93380 Insulin-like growth factor-I Meleagris gallopavo (Common turkey) 9.1×10-2
LVDALQ-FVCGDRGFYF B3GQC6 Insulin-like growth factor 1 Eospalax baileyi (Plateau zokor) (Myospalax baileyi) 9.1×10-2
LVDALQ-FVCGDRGFYF B3GQC7 Insulin-like growth factor 1 Ochotona curzoniae (Black-lipped pika) 9.1×10-2
LVDALQ-FVCGDRGFYF Q4JHY5 Insulin-like growth factor 1 Cervus nippon (Sika deer) 9.1×10-2
LVDALQ-FVCGDRGFYF A6NBZ1 Insulin-like growth factor 1 Bos gaurus frontalis (Domestic gayal) (Bos frontalis) 9.1×10-2
LVDALQ-FVCGDRGFYF D2IJT0 Insulin-like growth factor 1 Bubalus bubalis (Domestic water buffalo) 9.1×10-2
LVDALQ-FVCGDRGFYF P07455 Insulin-like growth factor I Bos taurus (Bovine) 9.1×10-2
LVDALQ-FVCGDRGFYF Q9N1C1 Insulin-like growth factor I Bos taurus (Bovine) 9.1×10-2
LVDALQ-FVCGDRGFYF D2DS56 Insulin like growth factor-1 Capra hircus (Goat) 9.1×10-2
LVDALQ-FVCGDRGFYF P51457 Insulin-like growth factor I Capra hircus (Goat) 9.1×10-2
LVDALQ-FVCGDRGFYF B5AMJ4 Insulin-like growth factor I Ovis aries (Sheep) 9.1×10-2
LVDALQ-FVCGDRGFYF A0FJ28 Insulin-like growth factor I Ovis aries (Sheep) 9.1×10-2
LVDALQ-FVCGDRGFYF P10763 Insulin-like growth factor I Ovis aries (Sheep) 9.1×10-2
LVDALQ-FVCGDRGFYF P10763-3 Isoform C Ovis aries (Sheep) 9.1×10-2
LVDALQ-FVCGDRGFYF P10763-2 Isoform A Ovis aries (Sheep) 9.1×10-2
LVDALQ-FVCGDRGFYF A0N8V2 Insuline-like growth factor-I (2) Ovis aries (Sheep) 9.1×10-2
LVDALQ-FVCGDRGFYF Q000X1 Insulin-like growth factor I Cervus elaphus (Red deer) 9.1×10-2
LVDALQ-FVCGDRGFYF Q45QB4 Insulin-like growth factor I Sus scrofa (Pig) 9.1×10-2
LVDALQ-FVCGDRGFYF P16545 Insulin-like growth factor I Sus scrofa (Pig) 9.1×10-2
LVDALQ-FVCGDRGFYF Q0PPT3 Class 1 insulin-like growth factor I Sus scrofa (Pig) 9.1×10-2
LVDALQ-FVCGDRGFYF Q0PPT2 Class 2 insulin-like growth factor I Sus scrofa (Pig) 9.1×10-2
LVDALQ-FVCGDRGFYF A7LHD8 Insulin-like growth factor 1 Equus caballus (Horse) 9.1×10-2
LVDALQ-FVCGDRGFYF Q6JLX1 Insulin-like growth factor I Ailuropoda melanoleuca (Giant panda) 9.1×10-2
LVDALQ-FVCGDRGFYF Q68LC0 Insulin-like growth factor I Pygathrix roxellana (Golden snub-nosed monkey) (Rhinopithecus roxellana) 9.1×10-2
LVDALQ-FVCGDRGFYF Q6IVA5 Insulin-like growth factor I Ailurus fulgens (Lesser panda) (Red panda) 9.1×10-2
LVDALQ-FVCGDRGFYF Q6GUL6 Insulin-like growth factor I Panthera tigris altaica (Siberian tiger) 9.1×10-2
LVDALQ-FVCGDRGFYF B7UCD1 Insulin-like growth factor I Mustela vison (American mink) (Neovison vison) 9.1×10-2
LVDALQ-FVCGDRGFYF Q9NP10 IGF1 protein Homo sapiens (Human) 9.1×10-2
LVDALQ-FVCGDRGFYF Q14620 Insulin-like growth factor 1 (Somatomedin C),... Homo sapiens (Human) 9.1×10-2
LVDALQ-FVCGDRGFYF Q5U743 Insulin-like growth factor 1 (Somatomedin C) Homo sapiens (Human) 9.1×10-2
LVDALQ-FVCGDRGFYF Q59GC5 Insulin-like growth factor 1 (Somatomedin C);... Homo sapiens (Human) 9.1×10-2
LVDALQ-FVCGDRGFYF P05019-2 Isoform 2 Homo sapiens (Human) 9.1×10-2
LVDALQ-FVCGDRGFYF B0FBP3 Insulin-like growth factor 1 Vulpes lagopus (Arctic fox) (Alopex lagopus) 9.1×10-2
LVDALQ-FVCGDRGFYF P33712 Insulin-like growth factor I Canis familiaris (Dog) (Canis lupus familiaris) 9.1×10-2
LVDALQ-FVCGDRGFYF P17647 Insulin-like growth factor I Cavia porcellus (Guinea pig) 9.1×10-2
LVDALQ-FVCGDRGFYF Q95222-2 Isoform IGF-IA Oryctolagus cuniculus (Rabbit) 9.1×10-2
LVDALQ-FVCGDRGFYF Q28933 Insulin-like growth factor I Suncus murinus (Asian house shrew) (Musk shrew) 9.1×10-2
LVDALQ-FVCGDRGFYF Q6GVY9 Insulin-like growth factor 1 Sus scrofa (Pig) 9.1×10-2
LVDALQ-FVCGDRGFYF Q95222 Insulin-like growth factor I Oryctolagus cuniculus (Rabbit) 9.1×10-2
LVDALQ-FVCGDRGFYF Q4JI74 Insulin-like growth factor 1 Bubalus bubalis (Domestic water buffalo) 9.1×10-2
LVDALQ-FVCGDRGFYF D2H1S1 Putative uncharacterized protein Ailuropoda melanoleuca (Giant panda) 9.1×10-2
LVDALQ-FVCGDRGFYF D8L628 Insulin-like growth factor I Sus scrofa (Pig) 9.1×10-2
LVDALQ-FVCGDRGFYF D8L627 Insulin-like growth factor I Sus scrofa (Pig) 9.1×10-2
LVDALQ-FVCGDRGFYF E2R442 Uncharacterized protein Canis familiaris (Dog) (Canis lupus familiaris) 9.1×10-2
LVDALQ-FVCGDRGFYF E7ESN8 Uncharacterized protein Homo sapiens (Human) 9.1×10-2
LVDALQ-FVCGDRGFYF P51458 Insulin-like growth factor I Equus caballus (Horse) 9.1×10-2
LVDALQ-FVCGDRGFYF P05019 Insulin-like growth factor I Homo sapiens (Human) 9.1×10-2
LVDALQ-FVCGDRGFYF Q13429 Insulin-like growth factor-I Homo sapiens (Human) 9.1×10-2
LVDALQ-FVCGDRGFYF Q6GVY8 Insulin-like growth factor 1 Sus scrofa (Pig) 9.1×10-2
LVDTLQ-FVCGERGFYF Q9YI57 Prepro-insulin-like growth factor I Acanthopagrus schlegelii (Black porgy) 9.1×10-2
LVDTLQ-FVCGERGFYF A5JSH0 Insulin-like growth factor I Sparus aurata (Gilthead sea bream) 9.1×10-2
LVDTLQ-FVCGERGFYF Q4G1F3 Insulin-like growth factor I Sparus aurata (Gilthead sea bream) 9.1×10-2
LVDTLQ-FVCGERGFYF B2ZRV7 Insulin-like growth factor I Hippoglossus hippoglossus (Atlantic halibut) (Pleuronectes hippoglossus) 9.1×10-2
LVDTLQ-FVCGERGFYF A6XBL3 Insulin-like growth factor-I Dentex dentex (common dentex) 9.1×10-2
LVDTLQ-FVCGERGFYF A8CG79 Preproinsulin-growth factor I Pagrus auriga 9.1×10-2
LVDTLQ-FVCGERGFYF A9XU11 Insulin-like growth factor 1 isoform b Sparus aurata (Gilthead sea bream) 9.1×10-2
LVDTLQ-FVCGERGFYF Q56V57 Insulin-like growth factor I Rhabdosargus sarba (goldlined seabream) 9.1×10-2
LVDTLQ-FVCGERGFYF A9XU12 Insulin-like growth factor 1 isoform c Sparus aurata (Gilthead sea bream) 9.1×10-2
LVDTLQ-FVCGERGFYF B0Z829 Insulin-like growth factor 1 Amphiprion clarkii (Clark's amemonefish) 9.1×10-2
LVDTLQ-FVCGERGFYF A2IX61 Insulin-like growth factor I Cottus kazika 9.1×10-2
LVDALQ-FVCGDRGFYF Q91442 Insulin-like growth factor I Squalus acanthias (Spiny dogfish) 9.1×10-2
LVDALQ-FVCGDRGFYF Q8AV14 Insulin-like growth factor Petromyzon marinus (Sea lamprey) 1.6×10-2
LVDTLQ-FVCDDRGFFF P22618 Insulin-like growth factor Myxine glutinosa (Atlantic hagfish) 5.0×10-2
