# Supplementary material for: Defining and searching for structural motifs using DeepView/Swiss-PdbViewer
Source: BMC Bioinformatics. 2012 Jul 23;13:173. doi: 10.1186/1471-2105-13-173 (PMC3436773; doi:10.1186/1471-2105-13-173)
Supplement: Additional file 3 — The motif specification created from pig insulin (pdb id 4ins), with delta-constraints between the second Leu and Tyr loosened by permitting deviations of ±25 from the corresponding sequence separation of the motif in pdb id 4ins. [file 1471-2105-13-173-S5.pdf]

Additional file 5 The (raw) results of CMEPS calculations of 2agk (see main text for citations) follow immediately below. Bold letters and digits are used for residues and values belonging to the motifs discussed in the text. Energies are in kcal/mol.

Residue	VdW	Elec	Desolv_elec	Desolv_np		DG_bind
THR2	-5.86	1.80	-0.03	-1.54	:	-5.64
LYS3	-6.75	-239.43	241.04	-2.28	:	-7.42
PHE4	-16.17	0.95	1.44	-2.31	:	-16.09
ILE5	-10.69	4.16	8.10	-2.05	:	-0.47
CYS7	-6.13	1.16	6.74	-1.55	:	0.23
ILE8	-10.65	4.94	0.73	-1.98	:	-6.96
ASP9	-3.79	54.37	-38.53	-1.57	:	10.47
LEU10	-11.36	3.80	2.32	-1.97	:	-7.21
HSE11	-5.23	-6.10	10.48	-1.71	:	-2.57
ASN12	-4.61	-6.46	10.29	-1.68	:	-2.46
GLU14	-4.03	11.12	2.42	-2.14	:	7.36
VAL15	-2.36	5.09	3.90	-1.54	:	5.09
LYS16	-7.15	-90.87	100.20	-2.20	:	-0.02
GLN17	-5.21	-0.07	7.49	-1.65	:	0.56
ILE18	-4.56	3.76	5.04	-1.83	:	2.41
VAL19	-1.06	4.95	2.16	-1.25	:	4.79
THR22	-4.10	-2.79	8.44	-1.62	:	-0.07
LEU23	-2.38	3.41	1.66	-1.35	:	1.35
THR24	-0.30	-3.56	6.51	-1.30	:	1.36
SER25	-0.36	-2.18	3.66	-1.23	:	-0.11
LYS26	-3.45	-33.40	40.56	-1.41	:	2.31
LYS27	-0.82	-135.31	135.47	-1.59	:	-2.24
GLU28	-2.51	6.07	2.01	-1.29	:	4.28
ASP29	0.98	-92.04	88.13	-1.55	:	-4.48
VAL30	-3.60	5.03	4.47	-1.68	:	4.23
LYS32	-2.69	-58.87	64.84	-1.32	:	1.96
THR33	-3.57	-3.37	8.51	-1.58	:	-0.00
ASN34	-5.75	-5.15	9.21	-1.65	:	-3.34
PHE35	-8.94	0.67	5.63	-2.27	:	-4.91
VAL36	-1.27	5.10	1.84	-1.37	:	4.30
SER37	-2.08	1.81	4.25	-1.33	:	2.66
GLN38	-3.26	5.06	2.76	-1.54	:	3.02
HSD39	-7.23	0.61	5.17	-1.84	:	-3.29
SER41	-1.97	-8.93	8.08	-1.29	:	-4.12
SER42	-3.18	-6.69	11.91	-1.36	:	0.68
TYR43	-10.42	0.99	4.31	-2.29	:	-7.40
TYR44	-16.29	-8.19	10.90	-2.59	:	-16.17
ALA45	-4.49	3.50	0.63	-1.13	:	-1.49
LYS46	-5.09	-188.78	193.68	-1.73	:	-1.91
LEU47	-9.02	2.69	3.10	-2.09	:	-5.32
TYR48	-18.24	-5.20	6.50	-2.50	:	-19.44
LYS49	-7.70	-176.03	182.78	-1.85	:	-2.79
ASP50	-2.78	40.64	-36.53	-1.42	:	-0.09
ARG51	-8.35	-211.89	217.45	-2.37	:	-5.15
ASP52	-2.11	66.75	-56.98	-1.38	:	6.27
VAL53	-9.44	5.64	1.26	-1.68	:	-4.22
GLN54	-4.96	-4.59	17.67	-1.84	:	6.28
CYS56	-9.26	1.68	1.01	-1.41	:	-7.98
HSD57	-11.86	1.15	8.12	-2.15	:	-4.74
VAL58	-8.38	5.69	0.05	-1.73	:	-4.36
ILE59	-8.10	3.25	5.28	-2.10	:	-1.67
LYS60	-9.84	-162.11	165.01	-2.37	:	-9.32
LEU61	-5.27	2.46	4.99	-1.89	:	0.30
ASN64	-3.40	-8.67	9.26	-1.64	:	-4.45

ASN65	-9.48	-10.24	10.42	-1.61	:	-10.91
ASP66	-1.39	-87.12	84.84	-1.58	:	-5.26
ASP67	-0.95	-43.46	42.73	-1.41	:	-3.09
ALA68	-3.89	4.06	-0.18	-1.15	:	-1.16
ALA69	-4.19	5.05	-0.64	-1.14	:	-0.92
ARG70	-10.13	-161.63	166.81	-2.38	:	-7.33
GLU71	-5.04	69.34	-51.09	-1.69	:	11.51
ALA72	-4.55	4.04	-0.93	-1.14	:	-2.57
LEU73	-12.05	4.25	-0.66	-1.97	:	-10.43
GLN74	-4.49	-1.90	10.16	-1.53	:	2.23
GLU75	-7.23	55.39	-43.33	-1.82	:	3.00
SER76	-4.35	-0.38	3.58	-1.29	:	-2.44
GLN78	-3.28	3.80	4.23	-1.47	:	3.29
PHE79	-14.54	-0.36	8.74	-2.47	:	-8.63
LEU80	-12.21	3.25	1.49	-1.97	:	-9.44
GLN81	-11.41	-12.52	20.85	-1.98	:	-5.06
VAL82	-8.75	4.99	0.30	-1.71	:	-5.16
ILE86	-12.02	3.48	4.43	-1.95	:	-6.06
ASN87	-5.16	-9.32	7.43	-1.60	:	-8.65
ASP88	-1.49	-84.50	83.87	-1.63	:	-3.74
THR89	-1.38	4.49	1.93	-1.27	:	3.77
ASN90	-5.17	-12.97	10.43	-1.66	:	-9.37
CYS91	-7.98	1.07	0.95	-1.41	:	-7.37
LEU92	-6.60	2.82	4.03	-1.89	:	-1.64
GLU93	-3.94	-33.10	41.38	-1.71	:	2.64
TRP94	-20.73	0.78	11.08	-2.97	:	-11.84
LEU95	-11.51	3.66	1.71	-1.99	:	-8.14
LYS96	-5.17	-136.68	140.74	-1.74	:	-2.85
TRP97	-15.24	1.95	8.77	-2.77	:	-7.28
ALA98	-4.62	4.62	-0.65	-1.13	:	-1.79
SER99	-2.12	-7.07	7.20	-1.36	:	-3.35
LYS100	-7.48	-193.70	194.27	-2.27	:	-9.19
VAL101	-9.60	6.33	-2.56	-1.67	:	-7.50
ILE102	-11.47	4.02	1.84	-1.94	:	-7.55
VAL103	-8.21	4.80	0.54	-1.67	:	-4.54
THR104	-2.17	-11.26	18.96	-1.77	:	3.77
SER105	-1.56	2.61	2.52	-1.23	:	2.33
TRP106	-18.96	-1.58	10.67	-2.89	:	-12.75
LEU107	-11.87	2.00	3.68	-1.98	:	-8.18
PHE108	-15.65	0.49	8.67	-2.55	:	-9.05
THR109	-3.92	-1.40	2.38	-1.63	:	-4.57
LYS110	-2.13	-47.99	53.52	-1.14	:	2.26
GLU111	-3.02	2.45	7.59	-1.37	:	5.65
HSD113	-5.70	-4.23	10.71	-1.76	:	-0.99
PHE114	-14.55	-0.86	6.95	-2.50	:	-10.96
GLN115	-7.42	1.02	7.91	-1.82	:	-0.30
LEU116	-7.79	1.71	5.33	-2.05	:	-2.80
LYS117	-3.56	-93.12	100.38	-1.36	:	2.34
ARG118	-11.87	-82.13	87.39	-2.32	:	-8.93
LEU119	-11.82	2.05	-2.29	-1.96	:	-14.02
GLU120	-5.12	-13.52	23.81	-1.79	:	3.39
ARG121	-6.34	-117.11	120.78	-2.09	:	-4.76
LEU122	-12.43	2.84	0.01	-2.01	:	-11.60
THR123	-5.41	-3.68	8.18	-1.62	:	-2.52
GLU124	-3.87	36.03	-26.26	-1.40	:	4.50
LEU125	-8.62	4.48	2.25	-2.00	:	-3.89
CYS126	-8.47	2.26	2.24	-1.41	:	-5.38
LYS128	-6.75	-144.05	152.52	-1.86	:	-0.14
ASP129	-1.13	-19.15	18.70	-1.42	:	-3.01
ARG130	-10.56	-158.10	165.85	-2.27	:	-5.08

ILE131	-12.27	5.16	1.91	-1.94	:	-7.15
VAL132	-8.06	5.21	-1.37	-1.67	:	-5.90
VAL133	-8.21	5.35	-1.99	-1.71	:	-6.56
ASP134	-4.87	23.57	-10.52	-1.78	:	6.41
LEU135	-10.84	3.83	1.13	-2.07	:	-7.95
SER136	-3.16	-5.46	10.11	-1.40	:	0.08
CYS137	-9.79	1.54	4.32	-1.41	:	-5.34
ARG138	-9.46	-129.72	135.03	-2.18	:	-6.32
LYS139	-7.76	-209.48	215.53	-2.21	:	-3.92
THR140	-3.62	-1.37	3.42	-1.60	:	-3.17
GLN141	-3.76	-5.95	13.85	-1.58	:	2.55
ASP142	-1.34	24.17	-21.61	-1.12	:	0.10
ARG144	-5.93	-178.09	177.90	-2.11	:	-8.22
TRP145	-22.38	-1.23	10.77	-2.83	:	-15.68
ILE146	-8.95	4.33	3.99	-2.02	:	-2.65
VAL147	-9.30	4.60	1.93	-1.69	:	-4.47
ALA148	-4.86	4.90	0.55	-1.14	:	-0.55
MET149	-12.04	3.63	3.33	-2.19	:	-7.27
ASN150	-3.67	-10.24	10.77	-1.56	:	-4.69
LYS151	-3.53	-99.04	106.38	-1.57	:	2.24
TRP152	-14.27	0.20	9.32	-2.79	:	-7.53
GLN153	-4.37	0.99	5.06	-1.45	:	0.23
THR154	-3.73	-2.92	7.54	-1.58	:	-0.68
LEU155	-8.06	3.23	3.05	-1.93	:	-3.72
THR156	-6.77	-3.48	2.65	-1.54	:	-9.14
ASP157	-1.27	-9.23	5.03	-1.54	:	-7.00
LEU158	-10.10	3.02	3.08	-2.06	:	-6.06
GLU159	-4.92	-15.68	22.45	-1.97	:	-0.12
LEU160	-11.33	2.92	-0.96	-1.96	:	-11.33
ASN161	-4.05	-20.22	19.44	-1.75	:	-6.58
ALA162	-2.77	4.16	2.23	-1.18	:	2.44
ASP163	-1.31	-9.92	11.51	-1.38	:	-1.09
THR164	-4.97	-2.39	6.65	-1.64	:	-2.35
PHE165	-16.47	2.25	1.28	-2.40	:	-15.35
ARG166	-5.93	-161.87	167.30	-1.97	:	-2.47
GLU167	-5.67	11.19	0.19	-1.73	:	3.98
LEU168	-10.33	2.84	1.29	-2.07	:	-8.28
ARG169	-10.34	-129.31	136.84	-2.33	:	-5.13
LYS170	-6.26	-134.01	140.99	-1.78	:	-1.06
TYR171	-13.78	-10.27	15.18	-2.61	:	-11.49
THR172	-5.37	-6.45	6.74	-1.54	:	-6.62
ASN173	-5.70	-8.43	14.83	-1.78	:	-1.07
GLU174	-5.10	-58.89	61.59	-1.96	:	-4.35
PHE175	-17.42	2.53	-0.64	-2.33	:	-17.87
LEU176	-10.35	2.90	5.27	-2.24	:	-4.42
ILE177	-11.76	4.70	0.06	-1.91	:	-8.91
HSE178	-7.46	-13.70	15.84	-2.02	:	-7.33
ALA179	-3.37	4.59	1.25	-1.22	:	1.25
ALA180	-3.05	5.73	0.02	-1.20	:	1.51
ASP181	-3.86	63.79	-43.12	-1.81	:	14.99
VAL182	-1.50	5.44	3.71	-1.49	:	6.16
GLU183	-3.45	58.00	-47.10	-1.49	:	5.96
LEU185	-1.83	3.45	1.62	-1.18	:	2.06
CYS186	-2.20	1.33	3.35	-1.18	:	1.30
ILE189	-9.95	4.64	3.05	-2.01	:	-4.27
ASP190	-3.02	-0.05	3.80	-1.73	:	-1.00
GLU191	-4.44	100.78	-88.87	-1.47	:	6.00
LEU192	-5.81	3.05	5.05	-1.88	:	0.41
**LEU193**	**-11.40**	**2.48**	**1.04**	**-2.03**	**:**	**-9.92**
**VAL194**	**-8.60**	**4.24**	**-0.32**	**-1.67**	**:**	**-6.35**

SER195	-3.07	-0.24	3.52	-1.31	:	-1.10
LYS196	-7.00	-201.43	200.07	-2.17	:	-10.54
**LEU197**	**-12.68**	**2.94**	**-1.44**	**-1.96**	**:**	**-13.14**
PHE198	-11.11	1.90	5.18	-2.43	:	-6.46
GLU199	-2.82	-18.11	22.08	-1.71	:	-0.56
TRP200	-21.37	3.09	6.93	-2.90	:	-14.26
THR201	-4.63	-7.21	6.24	-1.54	:	-7.15
LYS202	-6.91	-174.14	180.41	-1.88	:	-2.52
ASP203	-3.01	24.22	-15.84	-1.45	:	3.92
TYR204	-13.51	2.32	7.67	-2.65	:	-6.18
ASP205	-1.12	46.30	-42.05	-1.27	:	1.86
ASP206	-1.46	57.29	-52.67	-1.18	:	1.98
LEU207	-11.01	3.52	2.57	-2.04	:	-6.96
LYS208	-8.49	-175.90	193.15	-2.29	:	6.47
ILE209	-10.89	4.25	-0.26	-1.91	:	-8.81
VAL210	-8.00	5.53	2.54	-1.76	:	-1.69
**TYR211**	**-16.99**	**-0.44**	**7.00**	**-2.61**	**:**	**-13.03**
ALA212	-2.91	3.72	1.60	-1.20	:	1.21
ALA215	-3.72	3.68	0.73	-1.14	:	-0.44
LYS216	-6.23	-188.34	198.26	-2.29	:	1.39
SER217	-1.24	-13.03	11.78	-1.37	:	-3.86
VAL218	-4.73	5.08	1.37	-1.75	:	-0.04
ASP219	-2.14	55.70	-53.67	-1.30	:	-1.41
ASP220	-5.55	46.73	-42.01	-1.68	:	-2.50
LEU221	-13.24	3.38	-0.32	-1.96	:	-12.15
LYS222	-5.37	-213.63	217.56	-1.87	:	-3.31
LEU223	-6.84	1.94	4.39	-1.94	:	-2.46
VAL224	-8.81	5.15	-2.43	-1.67	:	-7.77
ASP225	-5.65	57.32	-43.65	-1.69	:	6.32
GLU226	-4.21	75.44	-63.25	-1.62	:	6.37
LEU227	-8.61	3.47	1.04	-1.95	:	-6.04
SER228	-3.87	-10.23	5.87	-1.29	:	-9.51
HSE229	-3.00	2.01	-1.92	-1.38	:	-4.29
LYS231	-9.47	-213.31	223.16	-2.37	:	-1.98
VAL232	-8.82	5.34	-0.38	-1.69	:	-5.55
ASP233	-5.94	12.15	-4.38	-1.73	:	0.10
LEU234	-13.03	2.29	-1.03	-1.93	:	-13.70
THR235	-6.39	-3.74	7.95	-1.63	:	-3.82
PHE236	-16.97	1.99	4.58	-2.33	:	-12.74
SER238	-1.35	2.76	2.10	-1.09	:	2.41
SER239	-3.43	-5.16	7.35	-1.31	:	-2.55
LEU240	-14.02	3.66	-0.41	-1.98	:	-12.74
ASP241	-1.89	2.74	-9.20	-1.59	:	-9.93
ILE242	-10.51	4.61	0.37	-1.99	:	-7.52
PHE243	-13.78	2.47	4.83	-2.45	:	-8.93
ASN246	-1.80	-4.35	5.17	-1.28	:	-2.26
LEU247	-5.76	3.24	3.46	-1.90	:	-0.95
VAL248	-7.42	4.32	1.60	-1.70	:	-3.19
LYS249	-5.87	-165.03	173.72	-1.69	:	1.13
PHE250	-15.60	1.21	4.09	-2.40	:	-12.70
GLU251	-3.03	14.65	-8.38	-1.68	:	1.57
ASP252	-4.14	11.14	-4.07	-1.61	:	1.32
CYS253	-9.32	1.80	4.38	-1.42	:	-4.57
CYS254	-8.73	0.90	3.52	-1.46	:	-5.77
ARG255	-5.87	-170.09	177.62	-1.81	:	-0.14
TRP256	-19.47	-3.24	9.60	-2.92	:	-16.04
ASN257	-7.67	-18.02	16.52	-1.80	:	-10.98
GLU258	-3.83	77.08	-66.92	-1.36	:	4.97
LYS259	-5.38	-154.48	161.67	-1.77	:	0.05
GLN260	-8.41	-7.50	19.35	-2.02	:	1.42
