# Supplementary material for: Defining and searching for structural motifs using DeepView/Swiss-PdbViewer
Source: BMC Bioinformatics. 2012 Jul 23;13:173. doi: 10.1186/1471-2105-13-173 (PMC3436773; doi:10.1186/1471-2105-13-173)
Supplement: Additional file 4 — Alignment of a fragment of chain B of insulin (4ins) and insulin-like proteins found in UniProtKB. The four residues participating in the structural motif discussed in the text and in Figure3are highlighted in bold. The three sequences which have a residue different than Tyr and Phe at the fourth position are underlined. [file 1471-2105-13-173-S6.pdf]

Additional file 6 The motif specification created from pdb id 2dpo, with distance constraints generated only for distances between Cα atoms, and with delta-constraints between motif-residues Ile and Ala as well as Ala and the last Val loosened by permitting deviations of ±25 from the corresponding sequence separation of the motif in pdb id 2dpo.

```
#SEARCH3D
# pattern defined from: 2DPO
# list of residues
# GroupNum allowed_kind allowed_Sec_Struct ; name chain num
GROUP     0 V      s   ; 'VAL' 'A' '9  '
GROUP     1 I      s   ; 'ILE' 'A' '11  '
GROUP     2 A      h   ; 'ALA' 'A' '22  '
GROUP     3 V      s   ; 'VAL' 'A' '32  '
GROUP     4 L      s   ; 'LEU' 'A' '34  '
# distances constraints
# (FromGrp FromAtom ToGrp ToAtom minDist optimalDist maxDist)
DIST     0  CA      1  CA         5.7    6.7    7.7
DIST     0  CA      2  CA         7.5    8.5    9.5
DIST     0  CA      3  CA         3.9    4.9    5.9
DIST     0  CA      4  CA         7.7    8.7    9.7
DIST     1  CA      2  CA         5.8    6.8    7.8
DIST     1  CA      3  CA         6.2    7.2    8.2
DIST     1  CA      4  CA         4.0    5.0    6.0
DIST     2  CA      3  CA         5.3    6.3    7.3
DIST     2  CA      4  CA         6.3    7.3    8.3
DIST     3  CA      4  CA         5.3    6.3    7.3
# backbone separation
# (FromGrp ToGrp min max)
DELTA     1     0      2      2
DELTA     2     1      1     36
DELTA     3     2      1     35
DELTA     4     3      2      2
# END
```
