# Supplementary material for: Defining and searching for structural motifs using DeepView/Swiss-PdbViewer
Source: BMC Bioinformatics. 2012 Jul 23;13:173. doi: 10.1186/1471-2105-13-173 (PMC3436773; doi:10.1186/1471-2105-13-173)
Supplement: Additional file 5 — The (raw) results of CMEPS calculations of 2agk (see main text for citations) follow immediately below. Bold letters and digits are used for residues and values belonging to the motifs discussed in the text. Energies are in kcal/mol. [file 1471-2105-13-173-S7.pdf]

Additional file 7 The (raw) results of computational alanine scanning of 1o94 using FoldX (see main text for citations) follow immediately below. Bold letters and digits are used for residues and values belonging to the motifs discussed in the text. Energies are in kcal/mol.

ALA1 0
ARG2 3.2155
ASP3 -0.115598
PRO4 1.18824
LYS5 -0.484904
HIS6 2.77824
ASP7 -0.131025
ILE8 1.19372
LEU9 3.3257
PHE10 3.56346
GLU11 0.102355
PRO12 1.29008
ILE13 2.7903
GLN14 0.674432
ILE15 4.28671
GLY16 1.29169
PRO17 1.66633
LYS18 2.78313
THR19 0.78136
LEU20 3.55255
ARG21 4.34525
ASN22 2.77701
ARG23 5.77567
PHE24 4.52905
TYR25 5.27501
GLN26 5.26426
VAL27 1.89813
PRO28 2.90916
HIS29 -0.809146
CYS30 -0.0832519
ILE31 3.05784
GLY32 1.31164
ALA33 0
GLY34 1.04527
SER35 0.972569
ASP36 -1.01904
LYS37 0.688259
PRO38 1.92917
GLY39 0.0537625
PHE40 2.8503
GLN41 5.01676
SER42 0.550748
ALA43 0
HIS44 -1.76547
ARG45 5.22307
SER46 0.49839
VAL47 1.636
LYS48 -4.25001
ALA49 0
GLU50 2.19033
GLY51 -0.0553937
GLY52 1.72216
TRP53 3.38451
ALA54 0
ALA55 0

LEU56 2.48504
ASN57 0.159292
THR58 2.34216
GLU59 4.44697
TYR60 5.08696
CYS61 0.29572
SER62 -0.327973
ILE63 3.17063
ASN64 0.662993
PRO65 2.61055
GLU66 2.28239
SER67 -0.202316
ASP68 3.26525
ASP69 2.45907
THR70 1.55881
HIS71 1.76265
ARG72 5.67191
LEU73 1.40151
SER74 0.148201
ALA75 0
ARG76 6.17718
ILE77 3.03112
TRP78 3.16235
ASP79 1.43841
GLU80 -0.609694
GLY81 -0.763234
ASP82 -1.57407
VAL83 1.44499
ARG84 3.67855
ASN85 2.32455
LEU86 2.47387
LYS87 1.15759
ALA88 0
MET89 2.13347
THR90 0.574582
ASP91 0.256958
GLU92 0.148266
VAL93 1.56508
HIS94 1.36774
LYS95 0.424172
TYR96 2.6519
GLY97 0.353217
ALA98 0
LEU99 2.29736
ALA100 0
GLY101 -0.658923
VAL102 2.60084
GLU103 -0.874444
LEU104 3.89742
TRP105 5.29077
TYR106 6.36811
GLY107 -0.0425385
GLY108 1.69907
ALA109 0
HIS110 5.08177
ALA111 0
PRO112 2.30084
ASN113 3.59907
MET114 -0.57952
GLU115 0.0648727

SER116 0.329896
ARG117 1.22623
ALA118 0
THR119 0.698936
PRO120 1.7948
ARG121 5.17058
GLY122 -0.376673
PRO123 3.05908
SER124 -0.994902
GLN125 0.814675
TYR126 3.4779
ALA127 0
SER128 1.98495
GLU129 0.973921
PHE130 2.32515
GLU131 1.35633
THR132 -0.371299
LEU133 -0.576649
SER134 -0.992837
TYR135 0.937868
CYS136 1.42205
LYS137 1.63179
GLU138 0.21676
MET139 2.8674
ASP140 3.29556
LEU141 0.155603
SER142 -0.00759235
ASP143 1.00797
ILE144 2.93023
ALA145 0
GLN146 1.04334
VAL147 2.0281
GLN148 1.36171
GLN149 0.522422
PHE150 2.10407
TYR151 3.60639
VAL152 1.11466
ASP153 -0.40609
ALA154 0
ALA155 0
LYS156 1.34094
ARG157 3.49327
SER158 -0.74328
ARG159 1.76115
ASP160 -0.806715
ALA161 0
GLY162 1.60678
PHE163 4.2639
ASP164 3.21319
ILE165 3.96042
VAL166 2.87795
TYR167 4.55696
VAL168 3.28349
TYR169 4.43656
GLY170 0.0364229
ALA171 0
HIS172 3.00083
SER173 -1.87045
TYR174 3.40975
LEU175 2.9914

PRO176	2.95251
LEU177	1.91544
GLN178	2.66558
PHE179	4.29439
LEU180	2.89882
ASN181	2.29629
PRO182	2.13505
TYR183	0.893356
TYR184	3.93087
ASN185	2.66121
LYS186	2.17695
ARG187	7.66878
THR188	-0.00569359
ASP189	4.74328
LYS190	0.716995
TYR191	5.58277
GLY192	-0.217067
GLY193	0.426292
SER194	1.0358
LEU195	2.51275
GLU196	0.716953
ASN197	0.87744
ARG198	4.3489
ALA199	0
ARG200	1.47302
PHE201	3.92795
TRP202	5.74322
LEU203	2.55853
GLU204	3.00479
THR205	0.781027
LEU206	3.53347
GLU207	-0.248511
LYS208	1.33746
VAL209	1.71409
LYS210	1.03423
HIS211	-0.42665
ALA212	0
VAL213	2.51675
GLY214	-0.73328
SER215	-0.29208
ASP216	-0.00809291
CYS217	0.385552
ALA218	0
ILE219	3.99315
ALA220	0
THR221	2.80447
ARG222	4.87966
PHE223	5.30563
GLY224	-1.10296
VAL225	1.65354
ASP226	-1.21618
THR227	0.815372
VAL228	0.504143
TYR229	1.53673
GLY230	0.787068
PRO231	0.510345
GLY232	0.325237
GLN233	1.72854
ILE234	2.73399
GLU235	-2.30919

ALA236	0
GLU237	-0.597385
VAL238	-1.1591
ASP239	0.575047
GLY240	0.200236
GLN241	0.913555
LYS242	0.119877
PHE243	4.88237
VAL244	2.32852
GLU245	-0.70461
MET246	1.96801
ALA247	0
ASP248	0.623054
SER249	-0.205768
LEU250	2.16786
VAL251	2.86698
ASP252	1.56922
MET253	1.82084
TRP254	6.41561
ASP255	2.24828
ILE256	4.27659
THR257	0.640022
ILE258	3.29186
GLY259	0.97655
ASP260	-0.847476
ILE261	2.25651
ALA262	0
GLU263	-0.337519
TRP264	4.14613
GLY265	-0.623756
GLU266	1.64947
ASP267	1.52583
ALA268	0
GLY269	-0.8028
PRO270	2.8368
SER271	-0.670636
ARG272	3.24211
PHE273	3.41017
TYR274	3.94734
GLN275	-0.538791
GLN276	0.655725
GLY277	1.63992
HIS278	-0.138793
THR279	-0.205736
ILE280	1.23531
PRO281	2.55339
TRP282	1.6929
VAL283	1.36274
LYS284	0.991441
LEU285	1.15641
VAL286	2.15188
LYS287	2.64728
GLN288	-0.806119
VAL289	1.49294
SER290	0.635326
LYS291	0.307331
LYS292	3.02105
PRO293	2.26405
VAL294	3.26401
LEU295	3.45963

GLY296	-0.919492
VAL297	0.729893
GLY298	0.0167337
ARG299	2.76094
TYR300	2.97783
THR301	0.291223
ASP302	3.80051
PRO303	2.6649
GLU304	0.0398975
LYS305	0.624391
MET306	2.75732
ILE307	1.61222
GLU308	0.721217
ILE309	2.36611
VAL310	1.63075
THR311	0.819434
LYS312	0.411084
GLY313	1.58144
TYR314	2.05185
ALA315	0
ASP316	3.04848
ILE317	4.85578
ILE318	3.34391
GLY319	-1.00272
CYS320	0.311388
ALA321	0
ARG322	2.19586
PRO323	2.69576
SER324	1.42519
ILE325	2.42815
ALA326	0
ASP327	7.54103
PRO328	3.02408
PHE329	4.13823
LEU330	2.46027
PRO331	2.11605
GLN332	0.770375
LYS333	3.81676
VAL334	2.10524
GLU335	0.349185
GLN336	0.773272
GLY337	1.80031
ARG338	2.71839
TYR339	3.60726
ASP340	-0.26873
ASP341	1.77484
ILE342	2.84444
ARG343	2.29198
VAL344	0.698053
CYS345	0.984911
ILE346	2.15858
GLY347	0.692613
CYS348	1.25704
ASN349	4.71808
VAL350	1.1529
CYS351	0.681713
ILE352	2.80265
SER353	0.291915
ARG354	1.18142
TRP355	4.35476

GLU356	0.969323
ILE357	0.988203
GLY358	0.631771
GLY359	0.22266
PRO360	2.16144
PRO361	1.10945
MET362	2.37159
ILE363	1.37068
CYS364	0.122413
THR365	0.918343
GLN366	2.08242
ASN367	3.27547
ALA368	0
THR369	0.325646
ALA370	0
GLY371	0.942081
GLU372	5.7092
GLU373	6.71387
TYR374	2.02915
ARG375	1.05672
ARG376	0.79945
GLY377	1.30988
TRP378	4.13261
HIS379	1.88816
PRO380	3.35476
GLU381	3.66394
LYS382	0.805384
PHE383	2.10423
ARG384	-0.00802767
GLN385	0.102637
THR386	1.22825
LYS387	-0.0626954
ASN388	0.327743
LYS389	0.536507
ASP390	-2.94935
SER391	1.22334
**VAL392**	**3.15414**
LEU393	3.95394
**ILE394**	**4.20079**
VAL395	2.76611
GLY396	1.19292
ALA397	0
GLY398	0.619528
PRO399	2.6593
SER400	-1.93191
GLY401	-0.702831
SER402	-0.850475
GLU403	8.94913
ALA404	0
**ALA405**	**0**
ARG406	0.230614
VAL407	1.56503
LEU408	3.12856
MET409	3.29048
GLU410	0.421957
SER411	-0.163544
GLY412	1.31298
TYR413	2.48831
THR414	0.261518
**VAL415**	**3.03327**

HIS416 2.05211
LEU417 3.91404
THR418 -0.680196
ASP419 1.24435
THR420 -0.468483
ALA421 0
GLU422 -0.230729
LYS423 0.260382
ILE424 2.91855
GLY425 2.0191
GLY426 0.88895
HIS427 0.247192
LEU428 2.64669
ASN429 1.9196
GLN430 -0.459145
VAL431 1.80836
ALA432 0
ALA433 0
LEU434 3.15262
PRO435 2.31928
GLY436 0.385889
LEU437 3.23744
GLY438 -0.692632
GLU439 2.12406
TRP440 2.81488
SER441 -0.469073
TYR442 1.16307
HIS443 0.78058
ARG444 6.68817
ASP445 0.661176
TYR446 2.99413
ARG447 3.04909
GLU448 1.8675
THR449 0.349857
GLN450 1.36568
ILE451 2.90761
THR452 0.194976
LYS453 1.29284
LEU454 0.0414218
LEU455 1.71158
LYS456 -0.101836
LYS457 0.237125
ASN458 1.07924
LYS459 -0.0572722
GLU460 -0.0722566
SER461 0.554139
GLN462 0.910756
LEU463 2.90007
ALA464 0
LEU465 2.96415
GLY466 1.1918
GLN467 0.0625432
LYS468 -0.171535
PRO469 0.591767
MET470 1.93812
THR471 2.49268
ALA472 0
ASP473 -0.702123
ASP474 1.88103
VAL475 2.81348

LEU476	2.5223
GLN477	0.288495
TYR478	1.72418
GLY479	0.790183
ALA480	0
ASP481	-0.738667
LYS482	2.8701
VAL483	3.44009
ILE484	4.04489
ILE485	4.35007
ALA486	0
THR487	0.674616
GLY488	1.66113
ALA489	0
ARG490	1.46928
TRP491	2.93375
ASN492	1.25031
THR493	0.0415043
ASP494	2.53422
GLY495	2.37657
THR496	2.17614
ASN497	2.82691
CYS498	0.0567454
LEU499	1.45438
THR500	1.54167
HIS501	0.445538
ASP502	-0.408462
PRO503	1.81857
ILE504	3.50073
PRO505	1.59499
GLY506	1.79612
ALA507	0
ASP508	2.50329
ALA509	0
SER510	2.25414
LEU511	0.272593
PRO512	1.83552
ASP513	-0.808223
GLN514	2.09039
LEU515	3.20659
THR516	1.38633
PRO517	3.54848
GLU518	1.30822
GLN519	2.12515
VAL520	2.16869
MET521	2.67374
ASP522	-0.873131
GLY523	1.84272
LYS524	0.302958
LYS525	2.64009
LYS526	-0.466048
ILE527	3.06822
GLY528	0.602198
LYS529	1.81901
ARG530	1.32855
VAL531	3.50869
VAL532	2.57026
ILE533	4.43545
LEU534	4.10277
ASN535	2.06593

ALA536 0
ASP537 -0.123065
THR538 -0.234033
TYR539 3.30096
PHE540 3.60312
MET541 3.32439
ALA542 0
PRO543 2.43441
SER544 1.14078
LEU545 3.69594
ALA546 0
GLU547 0.839266
LYS548 0.744868
LEU549 3.45137
ALA550 0
THR551 -0.0775168
ALA552 0
GLY553 1.92472
HIS554 2.38335
GLU555 0.388074
VAL556 3.58423
THR557 1.25057
ILE558 4.44698
VAL559 3.68345
SER560 0.0477018
GLY561 -1.03271
VAL562 1.81717
HIS563 -0.324161
LEU564 2.40625
ALA565 0
ASN566 -0.130586
TYR567 2.99779
MET568 2.37701
HIS569 -0.836755
PHE570 2.19376
THR571 1.91543
LEU572 3.70361
GLU573 2.12178
TYR574 1.72384
PRO575 1.3604
ASN576 1.7708
MET577 2.49421
MET578 1.8727
ARG579 2.58329
ARG580 0.976125
LEU581 3.36286
HIS582 -0.415492
GLU583 0.581308
LEU584 1.91257
HIS585 0.389731
VAL586 2.8181
GLU587 0.364918
GLU588 0.438041
LEU589 1.2766
GLY590 0.00753148
ASP591 0.540322
HIS592 0.599109
PHE593 4.50335
CYS594 1.42682
SER595 2.0516

ARG596	-0.0236396
ILE597	3.43253
GLU598	-0.518607
PRO599	0.424418
GLY600	1.69549
ARG601	0.619519
MET602	3.34122
GLU603	1.62268
ILE604	3.42688
TYR605	2.33463
ASN606	-0.780138
ILE607	1.17109
TRP608	0.516252
GLY609	-0.425866
ASP610	-0.133972
GLY611	-0.822901
SER612	-0.187511
LYS613	0.272644
ARG614	0.165607
THR615	-0.164117
TYR616	0.215853
ARG617	0.541683
GLY618	0.954328
PRO619	0.28254
GLY620	0.617978
VAL621	-0.00645554
SER622	0.382036
PRO623	0.904261
ARG624	0.959184
ASP625	0.642486
ALA626	0
ASN627	2.34679
THR628	0.0729859
SER629	1.1711
HIS630	1.84336
ARG631	0.455497
TRP632	1.75869
ILE633	2.16507
GLU634	-0.2647
PHE635	3.73716
ASP636	0.994297
SER637	1.09948
LEU638	4.00695
VAL639	3.84963
LEU640	3.37931
VAL641	2.26556
THR642	0.623879
GLY643	1.1658
ARG644	3.70839
HIS645	2.72432
SER646	-0.182067
GLU647	0.0823451
CYS648	-0.20165
THR649	-0.443265
LEU650	2.66075
TRP651	3.53835
ASN652	-0.532494
GLU653	-1.02893
LEU654	3.47266
LYS655	1.70804

ALA656	0
ARG657	3.08841
GLU658	-0.244848
SER659	-0.267377
GLU660	-0.816466
TRP661	4.55861
ALA662	0
GLU663	-1.59803
ASN664	0.331913
ASP665	-0.940765
ILE666	3.73839
LYS667	1.24508
GLY668	-0.349619
ILE669	4.82623
TYR670	3.1192
LEU671	3.37609
ILE672	2.94333
GLY673	1.609
ASP674	1.51733
ALA675	0
GLU676	0.963786
ALA677	0
PRO678	2.52911
ARG679	1.52451
LEU680	0.848141
ILE681	1.97858
ALA682	0
ASP683	1.73653
ALA684	0
THR685	0.828702
PHE686	1.42669
THR687	0.0194935
GLY688	-0.173134
HIS689	-1.23396
ARG690	3.3724
VAL691	1.38013
ALA692	0
ARG693	-1.51532
GLU694	3.13087
ILE695	2.0405
GLU696	-0.0126133
GLU697	-0.661597
ALA698	0
ASN699	1.09655
PRO700	2.17878
GLN701	1.49661
ILE702	0.666882
ALA703	0
ILE704	0.479671
PRO705	0.924724
TYR706	0.549804
LYS707	-0.0454529
ARG708	0.462165
GLU709	6.02034
THR710	-0.221743
ILE711	-1.31409
ALA712	0
TRP713	0.24576
GLY714	1.22999
THR715	0.245444

PRO716 0.475115
HIS717 -1.43855
MET718 -0.354666
PRO719 2.19934
GLY720 1.16366
GLY721 -0.592883
ASN722 1.61499
PHE723 1.09259
LYS724 -0.119074
ILE725 -0.322489
GLU726 1.03329
TYR727 0.389218
LYS728 0.267075
VAL729 -0.590856
