# Supplementary material for: Defining and searching for structural motifs using DeepView/Swiss-PdbViewer
Source: BMC Bioinformatics. 2012 Jul 23;13:173. doi: 10.1186/1471-2105-13-173 (PMC3436773; doi:10.1186/1471-2105-13-173)
Supplement: Additional file 6 — The motif specification created from pdb id 2dpo, with distance constraints generated only for distances between Cα atoms, and with delta-constraints between motif-residues Ile and Ala as well as Ala and the last Val loosened by permitting deviations of ±25 from the corresponding sequence separation of the motif in pdb id 2dpo. [file 1471-2105-13-173-S8.pdf]

Additional file 8 The (raw) results of computational alanine scanning of 2obk using FoldX (see main text for citations) follow immediately below. Bold letters and digits are used for residues and values belonging to the motifs discussed in the text. Energies are in kcal/mol.

ARG4 0.449513
LYS5 0.485107
PRO6 2.52087
GLU7 0.292143
VAL8 2.929
ILE9 1.9784
ILE10 3.96844
THR11 0.928979
TYR12 3.79396
CYS13 3.40187
THR14 0.412605
GLN15 -0.483799
CYS16 2.67571
GLN17 0.372981
TRP18 3.42312
LEU19 0.635092
LEU20 -0.356601
ARG21 1.42588
ALA22 0
ALA23 0
TRP24 1.09741
LEU25 3.24445
ALA26 0
GLN27 0.142708
GLU28 0.761626
LEU29 4.01581
LEU30 1.17406
SER31 0.256772
THR32 -0.665985
PHE33 3.0536
SER34 -0.507754
ASP35 -0.153324
ASP36 -0.94419
LEU37 4.24825
GLY38 -1.1853
LYS39 0.204018
VAL40 2.82404
SER41 -0.545701
LEU42 1.83342
GLU43 1.2865
PRO44 1.73374
ALA45 0
THR46 0.00740944
GLY47 -0.0718227
GLY48 0.968377
ALA49 0
PHE50 3.95932
ARG51 1.86679
ILE52 4.48577
THR53 0.0353804
CYS54 0.717001
ASP55 0.51127
GLY56 1.15037
VAL57 0.183323
GLN58 0.660547

ILE59 3.20486
TRP60 4.25932
GLU61 0.0918331
ARG62 2.18834
LYS63 -0.190521
ALA64 0
ASP65 1.12653
GLY66 0.478526
GLY67 0.426896
PHE68 3.15945
PRO69 2.66168
GLU70 0.243636
ALA71 0
LYS72 0.0828697
VAL73 0.951183
LEU74 3.18258
LYS75 0.553947
GLN76 -0.35254
ARG77 2.62393
VAL78 2.20813
ARG79 3.2557
ASP80 -0.485022
GLN81 -0.0494901
ILE82 1.70408
ASP83 -0.87081
PRO84 1.92179
GLU85 -0.862461
ARG86 0.368014
ASP87 -0.353759
