# Supplementary material for: Defining and searching for structural motifs using DeepView/Swiss-PdbViewer
Source: BMC Bioinformatics. 2012 Jul 23;13:173. doi: 10.1186/1471-2105-13-173 (PMC3436773; doi:10.1186/1471-2105-13-173)
Supplement: Additional file 7 — The (raw) results of computational alanine scanning of 4ins chain B using FoldX (see main text for citations) follow immediately below. Bold letters and digits are used for residues and values belonging to the motifs discussed in the text. Energies are in kcal/mol. [file 1471-2105-13-173-S9.pdf]

Additional file 9 The (raw) results of computational alanine scanning of 4ins chain B using FoldX (see main text for citations) follow immediately below. Bold letters and digits are used for residues and values belonging to the motifs discussed in the text. Energies are in kcal/mol.

PHE1 -0.846547
VAL2 -0.168376
ASN3 -0.119287
GLN4 0.517588
HIS5 2.04691
LEU6 2.75607
CYS7 3.58532
GLY8 0.906183
SER9 -0.689515
HIS10 6.08094
LEU11 2.73753
VAL12 0.0345413
GLU13 -0.349393
ALA14 0
LEU15 2.60775
TYR16 0.036318
LEU17 -0.358003
VAL18 1.25671
CYS19 2.23403
GLY20 0.657671
GLU21 -1.01228
ARG22 1.14802
GLY23 1.85037
PHE24 2.4151
PHE25 -0.831251
TYR26 2.54534
THR27 0.0956075
PRO28 1.77008
LYS29 1.01132
ALA30 0
