# Supplementary material for: Defining and searching for structural motifs using DeepView/Swiss-PdbViewer
Source: BMC Bioinformatics. 2012 Jul 23;13:173. doi: 10.1186/1471-2105-13-173 (PMC3436773; doi:10.1186/1471-2105-13-173)
Supplement: Additional file 8 — The (raw) results of computational alanine scanning of 2agk using FoldX (see main text for citations) follow immediately below. Bold letters and digits are used for residues and values belonging to the motifs discussed in the text. Energies are in kcal/mol. [file 1471-2105-13-173-S10.pdf]

Additional file 10 The (raw) results of computational alanine scanning of 2agk using FoldX (see main text for citations) follow immediately below. Bold letters and digits are used for residues and values belonging to the motifs discussed in the text. Energies are in kcal/mol.

```
THR2 0.917266
LYS3 1.55983
PHE4 4.72552
ILE5 2.65634
GLY6 -0.0349519
CYS7 0.00997166
ILE8 3.85976
ASP9 -0.255396
LEU10 3.51481
HIS11 0.215075
ASN12 0.344874
GLY13 1.70345
GLU14 0.479661
VAL15 1.18327
LYS16 0.661436
GLN17 -0.0921475
GLN38 -0.693291
HIS39 0.15282
PRO40 1.06779
SER41 -0.152703
SER42 -1.13674
TYR43 1.01997
TYR44 4.05083
ALA45 0
LYS46 0.276969
LEU47 2.036
TYR48 5.17271
LYS49 0.614406
ASP50 -1.01595
ARG51 1.96749
ASP52 -0.563745
VAL53 2.42561
GLN54 -0.389371
GLY55 0.795698
CYS56 2.49727
HIS57 2.45875
VAL58 3.0598
ILE59 2.62478
LYS60 1.33301
LEU61 0.906094
GLY62 1.08971
PRO63 0.720381
ASN64 0.579944
ASN65 1.58403
ASP66 0.698934
ASP67 -0.101783
ALA68 0
ALA69 0
ARG70 1.34881
GLU71 0.585891
ALA72 0
LEU73 3.3984
GLN74 0.311882
GLU75 -0.909972
SER76 -1.62095
```

PRO77 1.40872
GLN78 0.740501
PHE79 2.12383
LEU80 3.64646
GLN81 2.03909
VAL82 3.06871
GLY83 -0.517105
GLY84 1.55069
GLY85 1.17617
ILE86 2.73249
ASN87 1.01896
ASP88 -1.17882
THR89 -0.231559
ASN90 2.23648
CYS91 -0.0243666
LEU92 0.899072
GLU93 0.340349
TRP94 3.18074
LEU95 3.14779
LYS96 0.447712
TRP97 2.73494
ALA98 0
SER99 -1.54635
LYS100 1.87746
VAL101 2.66769
ILE102 4.13021
VAL103 1.65632
THR104 0.397618
SER105 -0.342966
TRP106 2.29399
LEU107 3.81515
PHE108 5.31856
THR109 0.93934
LYS110 0.162792
GLU111 1.1637
GLY112 1.49973
HIS113 1.16344
PHE114 4.25797
GLN115 0.164251
LEU116 0.717933
LYS117 -0.0674028
ARG118 1.4835
LEU119 3.54355
GLU120 -0.907995
ARG121 0.859574
LEU122 2.92879
THR123 0.254661
GLU124 -0.559861
LEU125 1.48329
CYS126 0.540404
GLY127 1.4203
LYS128 -0.243098
ASP129 0.307476
ARG130 3.87667
ILE131 3.08688
VAL132 3.10246
VAL133 3.20582
ASP134 0.560364
LEU135 3.67192
SER136 -0.220832

CYS137 0.636301
ARG138 1.98799
LYS139 1.20251
THR140 1.03589
GLN141 0.188222
ASP142 -0.0961623
GLY143 1.04998
ARG144 0.884333
TRP145 6.17411
ILE146 2.29199
VAL147 2.41091
ALA148 0
MET149 2.60046
ASN150 0.297128
LYS151 -0.0179529
TRP152 0.97633
GLN153 0.251406
THR154 1.05319
LEU155 1.33714
THR156 2.4993
ASP157 0.835838
LEU158 3.40651
GLU159 -0.203653
LEU160 3.74581
ASN161 1.85964
ALA162 0
ASP163 -0.385529
THR164 1.26898
PHE165 4.65303
ARG166 0.195008
GLU167 -0.0524769
LEU168 3.21656
ARG169 0.1011
LYS170 0.978489
TYR171 2.36508
THR172 2.7423
ASN173 -0.268189
GLU174 3.86912
PHE175 5.13477
LEU176 3.30993
ILE177 4.89164
HIS178 1.12702
ALA179 0
GLY187 0.0172475
GLY188 0.39669
ILE189 1.75737
ASP190 5.62461
GLU191 -0.557905
LEU192 0.22589
LEU193 3.06353
VAL194 2.18353
SER195 -0.232084
LYS196 1.28896
LEU197 3.98163
PHE198 2.23276
GLU199 -0.383503
TRP200 3.25012
THR201 1.66415
LYS202 1.49546
ASP203 -0.214035

TYR204	2.28993
ASP205	0.121268
ASP206	0.290665
LEU207	3.3742
LYS208	0.991773
ILE209	4.09843
VAL210	2.58734
**TYR211**	**4.29987**
ALA212	0
GLY213	-0.428759
GLY214	1.60942
ALA215	0
LYS216	0.224427
SER217	0.911818
VAL218	-0.143005
ASP219	-1.47158
ASP220	-1.14052
LEU221	3.17596
LYS222	1.06255
LEU223	1.38251
VAL224	2.66592
ASP225	-2.64435
GLU226	-1.03226
LEU227	2.47156
SER228	1.63244
HIS229	0.971105
GLY230	1.6614
LYS231	1.38754
VAL232	3.10531
ASP233	-0.553489
LEU234	3.68138
THR235	0.702495
PHE236	5.23806
GLY237	-0.361806
SER238	0.160388
SER239	-1.1017
LEU240	3.04771
ASP241	0.597766
ILE242	2.85762
PHE243	3.63741
GLY244	1.29836
GLY245	-0.29549
ASN246	0.428843
LEU247	0.998544
VAL248	2.85369
LYS249	0.170918
PHE250	4.52335
GLU251	-1.99082
ASP252	-1.49488
CYS253	-0.137042
CYS254	-0.424958
ARG255	0.812702
TRP256	3.6253
ASN257	1.61849
GLU258	-2.03042
LYS259	1.02266
GLN260	1.35593
GLY261	-0.112804
