# Supplementary material for: Defining and searching for structural motifs using DeepView/Swiss-PdbViewer
Source: BMC Bioinformatics. 2012 Jul 23;13:173. doi: 10.1186/1471-2105-13-173 (PMC3436773; doi:10.1186/1471-2105-13-173)
Supplement: Additional file 9 — The (raw) results of computational alanine scanning of 2dpo using FoldX (see main text for citations) follow immediately below. Bold letters and digits are used for residues and values belonging to the motifs discussed in the text. Energies are in kcal/mol. [file 1471-2105-13-173-S11.pdf]

Additional file 11 The (raw) results of computational alanine scanning of 2dpo using FoldX (see main text for citations) follow immediately below. Bold letters and digits are used for residues and values belonging to the motifs discussed in the text. Energies are in kcal/mol.

GLY7 -0.0335171
ASP8 1.67369
VAL9 3.16095
LEU10 4.11266
ILE11 3.85929
VAL12 2.63422
GLY13 0.624844
SER14 -0.547539
GLY15 1.63523
LEU16 -0.41131
VAL17 0.400278
GLY18 -1.00017
ARG19 1.69881
SER20 -0.261976
TRP21 5.09197
ALA22 0
MET23 2.71538
LEU24 1.61915
PHE25 4.48419
ALA26 0
SER27 -0.287073
GLY28 -1.29154
GLY29 1.15124
PHE30 2.61333
ARG31 1.29419
VAL32 3.55205
LYS33 3.40701
LEU34 3.40317
TYR35 2.24127
ASP36 2.81301
ILE37 -0.750479
GLU38 0.718325
PRO39 1.83449
ARG40 -0.0541307
GLN41 0.163864
ILE42 1.62871
THR43 0.224861
GLY44 -0.892148
ALA45 0
LEU46 1.37562
GLU47 0.374062
ASN48 -0.863592
ILE49 3.45723
ARG50 -0.329355
LYS51 0.264778
GLU52 0.380905
MET53 2.91471
LYS54 -0.614786
SER55 -0.763247
LEU56 1.93962
GLN57 -0.194684
GLN58 -0.410593
SER59 1.24083
GLY60 -0.262787
SER61 -0.603646

LEU62 2.20604
LYS63 -0.837218
GLY64 0.420233
SER65 -0.0611402
LEU66 0.583936
SER67 0.72303
ALA68 0
GLU69 0.28317
GLU70 0.653284
GLN71 0.116667
LEU72 1.86317
SER73 0.230588
LEU74 1.81032
ILE75 3.89749
SER76 -0.188089
SER77 -0.863897
CYS78 0.091442
THR79 0.236776
ASN80 1.28514
LEU81 2.30416
ALA82 0
GLU83 0.0784846
ALA84 0
VAL85 2.06954
GLU86 -0.190792
GLY87 0.983041
VAL88 2.67746
VAL89 0.489871
HIS90 2.26476
ILE91 4.68895
GLN92 3.31197
GLU93 5.41464
CYS94 -0.00774466
VAL95 2.43593
PRO96 1.95904
GLU97 1.7243
ASN98 1.97242
LEU99 2.10385
ASP100 -0.553785
LEU101 1.5578
LYS102 7.10475
ARG103 1.59488
LYS104 0.408764
ILE105 2.10101
PHE106 3.30906
ALA107 0
GLN108 -0.0224527
LEU109 3.58338
ASP110 3.62146
SER111 0.731952
ILE112 2.48923
VAL113 1.96478
ASP114 3.03743
ASP115 0.264073
ARG116 0.186552
VAL117 1.95938
VAL118 2.28035
LEU119 4.41664
SER120 -1.84151
SER121 1.13082

SER122 2.13414
SER123 0.811836
SER124 -0.408332
CYS125 0.903636
LEU126 2.1511
LEU127 1.37755
PRO128 3.39136
SER129 -0.0724663
LYS130 0.904351
LEU131 3.90956
PHE132 4.79791
THR133 1.62982
GLY134 0.731709
LEU135 2.1472
ALA136 0
HIS137 1.47764
VAL138 0.58847
LYS139 -0.015759
GLN140 1.90204
CYS141 0.511237
ILE142 3.32145
VAL143 2.68131
ALA144 0
HIS145 1.75474
PRO146 2.02893
VAL147 1.95615
ASN148 -0.324544
PRO149 2.61248
PRO150 2.75273
TYR151 1.70781
TYR152 3.42086
ILE153 2.76551
PRO154 1.21992
LEU155 2.27429
VAL156 3.13837
GLU157 4.91077
LEU158 3.4603
VAL159 3.5095
PRO160 1.69766
HIS161 4.08044
PRO162 1.67774
GLU163 0.686708
THR164 2.3803
SER165 1.69765
PRO166 1.31023
ALA167 0
THR168 0.338709
VAL169 1.21013
ASP170 0.820207
ARG171 1.2163
THR172 0.632724
HIS173 -2.41806
ALA174 0
LEU175 2.84243
MET176 3.05021
ARG177 -0.702464
LYS178 -0.0983749
ILE179 3.2337
GLY180 2.18862
GLN181 0.836001

SER182 -0.268175
PRO183 2.10882
VAL184 0.778072
ARG185 -0.681279
VAL186 2.24215
LEU187 -0.574534
LYS188 0.243323
GLU189 1.92993
ILE190 0.421688
ASP191 0.172451
GLY192 1.37193
PHE193 4.21212
VAL194 1.17534
LEU195 1.42183
ASN196 0.0765294
ARG197 1.25618
LEU198 0.040886
GLN199 0.668561
TYR200 0.686898
ALA201 0
ILE202 0.608061
ILE203 1.04947
SER204 -0.115431
GLU205 1.30212
ALA206 0
TRP207 3.01438
ARG208 2.92295
LEU209 0.888806
VAL210 1.73497
GLU211 0.090246
GLU212 -0.582439
GLY213 0.866682
ILE214 0.345491
VAL215 1.24444
SER216 0.661854
PRO217 2.44576
SER218 -0.82012
ASP219 -0.0174433
LEU220 1.63602
ASP221 -0.124041
LEU222 -0.198714
VAL223 -0.392624
MET224 1.10858
SER225 -0.0796464
ASP226 0.629681
GLY227 -0.270028
LEU228 0.789961
GLY229 -0.925976
MET230 -0.313838
ARG231 0.178911
TYR232 3.05959
ALA233 0
PHE234 2.61771
ILE235 1.13689
GLY236 -0.296451
PRO237 1.96378
LEU238 0.597959
GLU239 0.926068
THR240 0.107972
MET241 1.6388

HIS242 0.619195
LEU243 0.547686
ASN244 0.150458
ALA245 0
GLU246 0.311395
GLY247 1.94613
MET248 1.46415
LEU249 -0.466647
SER250 -1.60007
TYR251 2.75511
SER252 -0.889375
ASP253 0.207144
ARG254 0.168883
TYR255 1.70076
SER256 -0.898956
GLU257 0.312585
GLY258 -1.04758
MET259 0.840131
LYS260 -0.242878
ARG261 -0.248162
VAL262 -0.255124
LEU263 0.710803
LYS264 -0.379051
SER265 0.839381
PHE266 1.80335
GLY267 0.597849
SER268 -0.08907
ILE269 -0.209832
PRO270 2.40407
GLU271 -0.447727
PHE272 2.30029
SER273 -0.00649645
GLY274 1.74273
ALA275 0
THR276 1.57055
VAL277 -0.354089
GLU278 -0.167743
LYS279 0.655621
VAL280 1.40118
ASN281 -0.303887
GLN282 0.0306178
ALA283 0
MET284 0.404738
CYS285 0.282306
LYS286 -0.134813
LYS287 0.308573
VAL288 1.14381
PRO289 2.56807
ALA290 0
ASP291 5.14716
PRO292 1.87725
GLU293 -0.214301
HIS294 1.37009
LEU295 0.53392
ALA296 0
ALA297 0
ARG298 0.949598
ARG299 0.322997
GLU300 0.0129663
TRP301 0.279287

ARG302 0.896046
ASP303 1.13602
GLU304 1.25236
CYS305 0.666959
LEU306 1.74959
LYS307 -0.395977
ARG308 0.336849
LEU309 1.79875
ALA310 0
LYS311 0.433693
LEU312 0.859605
LYS313 1.90787
ARG314 -1.73879
GLN315 -0.00809642
MET316 -0.31468
