# Supplementary material for: Defining and searching for structural motifs using DeepView/Swiss-PdbViewer
Source: BMC Bioinformatics. 2012 Jul 23;13:173. doi: 10.1186/1471-2105-13-173 (PMC3436773; doi:10.1186/1471-2105-13-173)
Supplement: Additional file 10 — The (raw) results of computational alanine scanning of 1o94 using FoldX (see main text for citations) follow immediately below. Bold letters and digits are used for residues and values belonging to the motifs discussed in the text. Energies are in kcal/mol. [file 1471-2105-13-173-S1.pdf]

Additional file 1 A perl script (`make-spdbv-motif`) which takes a number of residue id:s (chain id and residue number concatenated into one word) and the name of a pdb-file as arguments and generates a motif specification involving the mentioned residues and the mentioned residues only.

The atom-types for which distance constraints are generated as well as the distance tolerances applied are selectable though command-line options. Constraints on secondary structure can be added, and constraints on amino-acid types and sequence separations loosened or changed by modifying the motif specifications generated by the script using a text editor. Information about options and arguments at various levels of detail are provided by typing the scripts name followed by one of the options `-man`, `-help` or `-usage`. As an illustration of the available options, we present the following example of a command-line:

```
make-spdbv-motif --atoms=C,CA,CB --uplim-factor=0.25 --uplim-const=0.0 \
    --lolim-factor=0.25 --lolim-const=0.0  A20 A22 A24 A25  1exr.pdb
```

which generates the following motif specification (shown in full in Additional file 2):

```
#SEARCH3D
# list of residues
# GroupNum allowed_kind allowed_Sec_Struct ; name chain num
GROUP     0 D      *   ; 'ASP' 'A' '20  '
GROUP     1 D      *   ; 'ASP' 'A' '22  '
GROUP     2 D      *   ; 'ASP' 'A' '24  '
GROUP     3 G      *   ; 'GLY' 'A' '25  '
# distance constraints
# (FromGrp FromAtom ToGrp ToAtom minDist optimalDist maxDist)
DIST      0   C      1   C         3.5    4.6    5.8
DIST      0   C      1  CA         3.3    4.4    5.6
DIST      0   C      1  CB         4.1    5.5    6.9
DIST      0  CA      1   C         4.2    5.7    7.1
DIST      0  CA      1  CA         4.2    5.6    7.0
DIST      0  CA      1  CB         4.9    6.5    8.1
DIST      0  CB      1   C         4.3    5.7    7.2
  :       :   :      :   :          :      :      :
DIST      1  CB      3  CA         6.5    8.7   10.8
DIST      2   C      3   C         2.4    3.2    4.0
DIST      2   C      3  CA         1.8    2.4    3.0
DIST      2  CA      3   C         3.4    4.6    5.7
DIST      2  CA      3  CA         2.9    3.8    4.8
DIST      2  CB      3   C         3.9    5.2    6.5
DIST      2  CB      3  CA         3.6    4.8    6.0
# backbone separation
# (FromGrp ToGrp min max)
DELTA     1     0     2     2
DELTA     2     1     2     2
DELTA     3     2     1     1
```

```
#!/usr/bin/env perl

=head1 NAME

make-spdbv-motif - Generate Swiss-PdbViewer motif files

=head1 SYNOPSIS

B<make-spdbv-motif> [I<OPTIONS>] I<resid1> I<resid2> ... I<residN> I<pdb-file>

=head1 DESCRIPTION

I<make-spdbv-motif> generates a motif specification for Swiss-PdbViewer from
an existing (PDB-format) three-dimensional protein structure file. Given a motif
specification, Swiss-PdbViewer can search individual PDB structures as well
as large collections of PDB structures for atom-configurations that satisfy
the specification.

The motif specification to generate is defined through the amino-acid residues
(given as the concatenation of chain id and residue number) that form the motif
in question and the PDB file containing the protein structure. The set of
atom-types between which distance constraints are generated is governed by
command-line options.

The generated motif specification is written to standard output.

=head1 OPTIONS

The following options are supported:

=over 4

=item B<--atoms>=I<ATOMLIST>

=over 2

Generate distance constraints between pairs of atom-types named
in I<ATOMLIST> (a comma-separated list of atom-names). The default
value used is the list "N,C,CA".

=back

=item B<--excl-atompairs>=I<PAIRLIST>

=over 2

Exclude the pairs of atom-types enumerated in I<PAIRLIST> from the
pairs of atom-types for which distance constraints are generated.
The default value is the empty list.

=back

=item B<--incl-atompairs>=I<PAIRLIST>

=over 2

Include the pairs of atom-types enumerated in I<PAIRLIST> in the
pairs of atom-types for which distance constraints are generated,
in addition to those implied by the --atoms option. Note that
atom-pair exclusion (using --excl-atompairs) has priority over
atom-pair inclusion. The default value is the empty list.

=back

=item B<--intra-residue>

=over 2

Include pairs of atoms belonging to the same residue
among those for which distance constraints are generated.

=back

=item B<--no-intra-residue>

=over 2
```

Exclude pairs of atoms belonging to the same residue
from those for which distance constraints are generated.

=back

=item B<--uplim-factor>=I<FLOAT-VALUE>

=over 2

Factor, multiplied by and added to each distance in question, to
obtain upper limit tolerances for distance constraints
(default value: 0.1).

=back

=item B<--lolim-factor>=I<FLOAT-VALUE>

=over 2

Factor,multiplied by and subtracted from each distance in question,
to obtain lower limit tolerances for distance constraints
(default value: 0.1).

=back

=item B<--uplim-const>=I<FLOAT-VALUE>

=over 2

Constant added to each corresponding distance, to obtain
upper limit tolerances for distance constraints
(default: 1.0).

=back

=item B<--lolim-const>=I<FLOAT-VALUE>

=over 2

Constant subtracted from each corresponding distance, to
obtain lower limit tolerances for distance constraints
(default: 1.0).

=back

=item B<--kill-dist>=I<FLOAT-VALUE>

=over 2

Cutoff distance. Dinstance constraints are not generated
between atoms between which the distance in the pdb-file
exceeds the cutoff distance (default: 15.0).

=back

=head1 EXAMPLES

The command:

=item C<make-spdbv-motif A20 A22 A24 A25 pdb1exr.ent>

=back

generates a motif specification corresponding to the first
of the so-called EF-hands in Calmodulin (comprising Asp20,
Asp22, asp24 and Gly25, in chain A), under the assumption
that the file pdb1exr.ent contains the PDB-format coordinates
of Calmodulin (pdb id: 1exr).

The command:

=item C<make-spdbv-motif --incl-atompairs=OG:CE1,OG:NE2,ND1:OD1,ND1:OD2 \ --excl-atompairs=CA:CB --
atoms=CA,CB A57 A102 A195 pdb1a0j.ent>

=back

```
generates a motif specification for the His/Asp/Ser catalytic
triad of trypsin from Atlantic salmon (pdb id: 1a0j), with distance
contraints between all combinations of CA and CB atoms and between
OG-CE1, OG-NE2, ND1-OD1 and ND1-OD2 atoms, located in His57, Asp102
or Ser195 (in chain A). Note that in order not to restrict the a
search that uses the generated motif, to exactly the sequence
separations between His57, Asp102 and Ser195, the last two lines
of the motif specification must be appropriately modified.

=head1 BUGS
Motifs spanning multiple chains are currently not properly handled.
The option C<--intra-residues> is not yet implemented.
Does not read compressed (e.g., gzipped) pdb-format files.

=head1 AUTHOR

Maria U. Johansson, Swiss Institute of Bioinformatics, Lausanne, Switzerland.

=head1 COPYRIGHT and LICENSE

This program is free and open software, You may use, modify, distribute,
and sell this program (and any modified variants) in any way you wish,
provided you do not restrict others from doing the same.

=cut

use File::Basename;
use Getopt::Long;
use Pod::Usage;
use strict;

sub Usage {
    my($prog)=(fileparse($0,'\..*'))[0];
    die <<EOF
Usage: $prog [options] resid1 resid2 ... residN pdb-file
EOF
}

sub max {
    my $a = shift;
    my $b = shift;
    return $a > $b ? $a : $b;
}

sub min {
    my $a = shift;
    my $b = shift;
    return $a < $b ? $a : $b;
}

my $atoms_optval = "N,C,CA";
my $excl_atompairs_optval = "";
my $incl_atompairs_optval = "";
my $intra_residue_optval = 0;
my $uplim_factor = 0.10;
my $lolim_factor = 0.10;
my $uplim_const = 1.0;
my $lolim_const = 1.0;
my $kill_dist = 15.0;
my $usage = 0;
my $help = 0;
my $man = 0;
GetOptions( "atoms=s" => \$atoms_optval,
            "excl-atompairs=s" => \$excl_atompairs_optval,
            "incl-atompairs=s" => \$incl_atompairs_optval,
            "intra-residue!" => \$intra_residue_optval,
            "uplim-factor=f" => \$uplim_factor,
            "lolim-factor=f" => \$lolim_factor,
            "uplim-const=f" => \$uplim_const,
            "lolim-const=f" => \$lolim_const,
            "kill-dist=f" => \$kill_dist,
            "usage" => \$usage, "help|?" => \$help, "man" => \$man );
if ( $usage ) {
    Usage();
}
```

```
pod2usage(1) if $help;
pod2usage(-existstatus => 0, -verbose=>2) if $man;

my @pairs = ( );
my %inclpairs = ( );
my %exclpairs = ( );
my %atomhash = ( );
my @atoms = split( /,/, $atoms_optval );
foreach my $a (@atoms) {
    $atomhash{$a} = 1;
}
foreach my $p ( split(/,/,$incl_atompairs_optval) ) {
    if ( $p =~ s/[\-:]/:/osg ) {
        $inclpairs{$p} = 1;
    } else {
        my($prog)=(fileparse($0,'\..*'))[0];
        die( "$prog: Invalid atom-pair specification: '$p'\n" );
    }
}
foreach my $p ( split(/,/,$excl_atompairs_optval) ) {
    if ( $p =~ s/[\-:]/:/osg ) {
        $exclpairs{$p} = 1;
        my @parts = split( /:/, $p );
        $exclpairs{$parts[1] . ":" . $parts[0]} = 1;
    } else {
        my($prog)=(fileparse($0,'\..*'))[0];
        die( "$prog: Invalid atom-pair specification: '$p'\n" );
    }
}
my %resids = ( );
my $grpcnt = 0;
while ( @ARGV > 1 ) {
    my $rid = shift(@ARGV);
    $resids{$rid} = $rid;
    $grpcnt += 1;
}
if ( ! -r $ARGV[0] ) {
    my($prog)=(fileparse($0,'\..*'))[0];
    die( "$prog: Can't read '$ARGV[0]': $!\n" );
}
my %abbrmap = ( "GLY" => "G", "ALA" => "A", "VAL" => "V", "LEU" => "L",
                "ILE" => "I", "MET" => "M", "PHE" => "F", "TRP" => "W",
                "PRO" => "P", "SER" => "S", "THR" => "T", "CYS" => "C",
                "TYR" => "Y", "ASN" => "N", "GLN" => "Q", "ASP" => "D",
                "GLU" => "E", "LYS" => "K", "ARG" => "R", "HIS" => "H" );
my %posx = ( );
my %posy = ( );
my %posz = ( );
my @groups = ( );
my $currid = "Z" . (128 * 256 * 256 * 256 - 1);
while ( <> ) {
    if ( /^ATOM/ ) {
        chomp;
        my $line = $_;
        my $chainstr = substr( $line, 21, 1 );
        my $seqno = 1 * substr( $line, 22, 5 );
        my $rid = $chainstr . $seqno;
        if ( exists $resids{$rid} ) {
            if ( $rid ne $currid ) {
                my @restyp = split( ' ', substr($line,16,4) );
                push( @groups, [$chainstr,$seqno,$restyp[0]] );
                $currid = $rid;
            }
            my @atm = split( ' ', substr($line,12,4) );
            my $idx = $rid . ":" . $atm[0];
            $posx{$idx} = 1.0 * substr( $line, 30, 8 );
            $posy{$idx} = 1.0 * substr( $line, 38, 8 );
            $posz{$idx} = 1.0 * substr( $line, 46, 8 );
        }
    }
}

if ( @groups != $grpcnt ) {
    my($prog)=(fileparse($0,'\..*'))[0];
    die( "$prog: not all argument residues are present in the structure\n" );
}
```

```
printf( "#SEARCH3D\n# list of residues\n" );
printf( "# GroupNum allowed_kind allowed_Sec_Struct ; name chain num\n" );
for ( my $j = 0 ; $j < @groups ; $j++ ) {
    my $chn = $groups[$j][0];
    my $seqno = $groups[$j][1];
    my $restyp = $groups[$j][2];
    if ( ! (exists $abbrmap{$restyp}) ) {
       my($prog)=(fileparse($0,'\..*'))[0];
       die( "$prog: Unrecognized residue-type '$restyp' in structure\n");
    }
    printf( "GROUP  %3d %1s      *    ", $j, $abbrmap{$restyp} );
    printf( "; '%s' '%s' '%-4d'\n", $restyp, $chn, $seqno );
}
printf( "# distance constraints\n" );
printf( "# (FromGrp FromAtom ToGrp ToAtom minDist optimalDist maxDist)\n" );
my @reslst = sort { substr($a,1) <=> substr($b,1) } keys( %resids );
my $count = $#reslst + 1;
for ( my $j = 0 ; $j < $count ; $j++ ) {
    my $rjn = $reslst[$j];
    for ( my $k = $j+1 ; $k < $count ; $k++ ) {
       my $rkn = $reslst[$k];
       foreach my $aj (@atoms) {
           my $idxj = $rjn . ":" . $aj;
           if ( exists $posx{$idxj} ) {
              foreach my $ak (@atoms) {
                  if ( ! exists($exclpairs{$aj . ":" . $ak}) ) {
                     my $idxk = $rkn . ":" . $ak;
                     if ( exists $posx{$idxk} ) {
                         my $dx = $posx{$idxj} - $posx{$idxk};
                         my $dy = $posy{$idxj} - $posy{$idxk};
                         my $dz = $posz{$idxj} - $posz{$idxk};
                         my $dist = sqrt( $dx*$dx + $dy*$dy + $dz*$dz );
                         if ( $dist <= $kill_dist ) {
                            printf( "DIST %5d %3s  %5d %3s      ", $j, $aj, $k, $ak );
                            my $lolim = &min($dist-$lolim_factor*$dist,$dist-$lolim_const);
                            my $uplim = &max($dist+$uplim_factor*$dist,$dist+$uplim_const);
                            printf( "%5.1f  %5.1f  %5.1f\n", $lolim, $dist, $uplim );
                         }
                     }
                  }
              }
           }
       }
       foreach my $ajk (keys %inclpairs) {
           if ( ! exists($exclpairs{$ajk}) ) {
              my @parts = split( /:/, $ajk );
              if ( ! (exists($atomhash{$parts[0]}) && exists($atomhash{$parts[1]})) ) {
                  my $idxj = $rjn . ":" . $parts[0];
                  my $idxk = $rkn . ":" . $parts[1];
                  if ( exists $posx{$idxj} && exists $posx{$idxk} ) {
                     my $dx = $posx{$idxj} - $posx{$idxk};
                     my $dy = $posy{$idxj} - $posy{$idxk};
                     my $dz = $posz{$idxj} - $posz{$idxk};
                     my $dist = sqrt( $dx*$dx + $dy*$dy + $dz*$dz );
                     if ( $dist <= $kill_dist ) {
                         printf( "DIST %5d %3s  %5d %3s      ", $j, $parts[0], $k, $parts[1] );
                         my $lolim = &min($dist-$lolim_factor*$dist,$dist-$lolim_const);
                         my $uplim = &max($dist+$uplim_factor*$dist,$dist+$uplim_const);
                         printf( "%5.1f  %5.1f  %5.1f\n", $lolim, $dist, $uplim );
                     }
                  }
                  if ( $parts[1] ne $parts[0] ) {
                     my $idxj = $rjn . ":" . $parts[1];
                     my $idxk = $rkn . ":" . $parts[0];
                     if ( exists $posx{$idxj} && exists $posx{$idxk} ) {
                         my $dx = $posx{$idxj} - $posx{$idxk};
                         my $dy = $posy{$idxj} - $posy{$idxk};
                         my $dz = $posz{$idxj} - $posz{$idxk};
                         my $dist = sqrt( $dx*$dx + $dy*$dy + $dz*$dz );
                         if ( $dist <= $kill_dist ) {
                            printf( "DIST %5d %3s  %5d %3s      ", $j, $parts[1], $k, $parts[0] );
                            my $lolim = &min($dist-$lolim_factor*$dist,$dist-$lolim_const);
                            my $uplim = &max($dist+$uplim_factor*$dist,$dist+$uplim_const);
                            printf( "%5.1f  %5.1f  %5.1f\n", $lolim, $dist, $uplim );
                         }
                     }
```

```
                    }
                }
            }
        }
    }
}
printf( "# backbone separation\n" );
printf( "# (FromGrp ToGrp min max)\n" );
@reslst = sort { $a <=> $b } map( substr($_,1), @reslst );
for ( my $j = 1 ; $j < $count ; $j++ ) {
    my $diff = 1*$reslst[$j] - 1*$reslst[$j-1];
    printf( "DELTA  %4d  %4d  %5d  %5d\n", $j, $j-1, $diff, $diff );
}

__END__
```
