# Supplementary material for: Defining and searching for structural motifs using DeepView/Swiss-PdbViewer
Source: BMC Bioinformatics. 2012 Jul 23;13:173. doi: 10.1186/1471-2105-13-173 (PMC3436773; doi:10.1186/1471-2105-13-173)
Supplement: Additional file 11 — The (raw) results of computational alanine scanning of 2obk using FoldX (see main text for citations) follow immediately below. Bold letters and digits are used for residues and values belonging to the motifs discussed in the text. Energies are in kcal/mol. [file 1471-2105-13-173-S2.pdf]

Additional file 2 A DxDxDG motif specification created by the script in Additional file 1.

```
#SEARCH3D
# list of residues
# GroupNum allowed_kind allowed_Sec_Struct ; name chain num
GROUP     0 D      *   ; 'ASP' 'A' '20  '
GROUP     1 D      *   ; 'ASP' 'A' '22  '
GROUP     2 D      *   ; 'ASP' 'A' '24  '
GROUP     3 G      *   ; 'GLY' 'A' '25  '
# distance constraints
# (FromGrp FromAtom ToGrp ToAtom minDist optimalDist maxDist)
DIST     0  C       1  C     3.4   4.6   5.8
DIST     0  C       1  CA    3.3   4.4   5.5
DIST     0  C       1  CB    4.1   5.5   6.9
DIST     0  CA      1  C     4.3   5.7   7.1
DIST     0  CA      1  CA    4.2   5.6   7.0
DIST     0  CA      1  CB    4.9   6.5   8.1
DIST     0  CB      1  C     4.3   5.7   7.1
DIST     0  CB      1  CA    4.6   6.1   7.6
DIST     0  CB      1  CB    5.2   7.0   8.8
DIST     0  C       2  C     5.4   7.2   9.0
DIST     0  C       2  CA    5.1   6.8   8.5
DIST     0  C       2  CB    5.5   7.3   9.1
DIST     0  CA      2  C     5.2   6.9   8.6
DIST     0  CA      2  CA    5.2   6.9   8.6
DIST     0  CA      2  CB    5.6   7.4   9.2
DIST     0  CB      2  C     4.4   5.9   7.4
DIST     0  CB      2  CA    4.6   6.1   7.6
DIST     0  CB      2  CB    5.1   6.8   8.5
DIST     0  C       3  C     5.6   7.4   9.2
DIST     0  C       3  CA    5.5   7.3   9.1
DIST     0  CA      3  C     4.9   6.5   8.1
DIST     0  CA      3  CA    5.0   6.7   8.4
DIST     0  CB      3  C     4.0   5.3   6.6
DIST     0  CB      3  CA    4.0   5.3   6.6
DIST     1  C       2  C     4.3   5.7   7.1
DIST     1  C       2  CA    3.4   4.5   5.6
DIST     1  C       2  CB    3.6   4.8   6.0
DIST     1  CA      2  C     5.1   6.8   8.5
DIST     1  CA      2  CA    4.2   5.6   7.0
DIST     1  CA      2  CB    4.3   5.7   7.1
DIST     1  CB      2  C     5.2   6.9   8.6
DIST     1  CB      2  CA    4.3   5.7   7.1
DIST     1  CB      2  CB    4.0   5.3   6.6
DIST     1  C       3  C     5.9   7.9   9.9
DIST     1  C       3  CA    5.4   7.2   9.0
DIST     1  CA      3  C     6.6   8.8  11.0
DIST     1  CA      3  CA    6.2   8.3  10.4
DIST     1  CB      3  C     6.8   9.0  11.2
DIST     1  CB      3  CA    6.5   8.7  10.9
DIST     2  C       3  C     2.4   3.2   4.0
DIST     2  C       3  CA    1.8   2.4   3.0
DIST     2  CA      3  C     3.4   4.6   5.8
DIST     2  CA      3  CA    2.8   3.8   4.8
DIST     2  CB      3  C     3.9   5.2   6.5
DIST     2  CB      3  CA    3.6   4.8   6.0
# backbone separation
# (FromGrp ToGrp min max)
DELTA     1     0      2      2
DELTA     2     1      2      2
DELTA     3     2      1      1
```
